# Fine‐Scale Variation in Soil Properties Promotes Local Taxonomic Diversity of Hybridizing Oak Species (*Quercus* spp.)

**DOI:** 10.1111/eva.70076

**Published:** 2025-02-06

**Authors:** Felix Zimmermann, Oliver Reutimann, Andri Baltensweiler, Lorenz Walthert, Jill K. Olofsson, Christian Rellstab

**Affiliations:** ^1^ Swiss Federal Research Institute WSL Birmensdorf Switzerland; ^2^ Institute of Integrative Biology ETH Zurich Zurich Switzerland; ^3^ Section for Forest and Landscape Ecology, Department of Geosciences and Natural Resource Management University of Copenhagen Kobenhavn Denmark

**Keywords:** adaptation, drought, hybridization, oaks, *Quercus*, soil

## Abstract

Although many tree species frequently hybridize and backcross, management decisions in forestry and nature conservation are usually concentrated on pure species. Therefore, understanding which environmental factors drive the distribution and admixture of tree species on a local stand scale is of great interest to support decision‐making in the establishment and management of resilient forests. Here, we extensively sampled a mixed stand of hybridizing white oaks ( *Quercus petraea* and *Q. pubescens*) near Lake Neuchâtel (Switzerland), where limestone and glacier moraine geologies coexist in proximity, to test whether micro‐environmental conditions can predict taxonomic distribution and genetic admixture. We collected DNA from bud tissue, individual soil samples, and extracted high‐resolution topographic data for 385 oak trees. We used 50 species‐discriminatory single nucleotide polymorphism (SNP) markers to determine the taxonomic composition and admixture levels of individual trees and tested their association with micro‐environmental conditions. We show that the trees' taxonomic distribution can be explained mainly by geographic position, soil pH, and potential rooting depth, a proxy for soil water availability. We found that admixed individuals tend to grow in habitats that are characteristic of the more drought‐tolerant species *Q. pubescens* rather than in intermediate habitats. Using in situ measurements, we are the first to show that fine‐scale variation in soil properties related to pH and water availability potentially drives the distribution of hybridizing tree species in a mixed stand. Microenvironmental variation therefore promotes local taxonomic diversity, facilitates admixture and adaptive introgression, and contributes to the resilience of forests under environmental change. Consequently, species such as white oaks should be managed and protected as a species complex rather than as pure species.

## 1 Introduction

The study of the distribution of plant species finds its roots in the pioneering work of von Humboldt and Bonpland (1850). Since then, this field has evolved significantly, culminating in broadly applied species distribution models (SDMs; Qazi, Saqib, and Zaman‐ul‐Haq 2022). These models predict species distributions in a statistical and probabilistic fashion based on modeled areas of suitable habitats across large spatial scales (Guisan and Zimmermann 2000). The distribution ranges of species can be explained by a variety of factors such as climate, soil properties, and anthropogenic impacts (Chauvier et al. 2021; Pellissier et al. 2013; Woodward and Williams 1987). Amidst the challenges posed by climate change, SDMs emerge as valuable tools capable of predicting species distribution ranges under future conditions. Incorporating relevant predictors at high resolution into SDMs can increase the quality of predictions and thereby deepen our understanding of species distribution dynamics (Austin and Van Niel 2011). Challenges persist in acquiring ecologically meaningful predictors, such as the modeling of soil properties due to their spatial heterogeneity (Vereecken et al. 2016). Consequently, soil properties are often absent in SDMs (Roe et al. 2022). Since classical SDMs treat species as homogenous units, the incorporation of intraspecific genetic variation, such as evolutionary lineages, adaptive differences, subspecies, or admixture and hybridization, into SDMs could further enhance the accuracy of predicting a species' potential habitat under future climate scenarios (Aguirre‐Liguori, Ramirez‐Barahona, and Gaut 2021; Lecocq et al. 2019; Razgour et al. 2019; Waldvogel et al. 2020).

Investigating the factors influencing species distributions on a local scale can contribute to a better understanding of species dynamics. Studies on different species, including hybridizing species complexes, have shown an effect of multiple abiotic and biotic factors on local distribution patterns (Bátori et al. 2017; Cosham, Beazley, and McCarthy 2016; Potts et al. 2020). A well‐studied example is *Irises* (Arnold et al. 2010). The distributions of different *Iris* taxa are, among other things, related to the microenvironment (Johnston et al. 2001). Thus, some *Iris* species have distinct ecological niches, but pure and admixed individuals can also be found in sympatry (Cruzan and Arnold 1993). Although it is known that tree species show small‐scale, intraspecific patterns of environmental adaptation (Budde et al. 2024; Scotti et al. 2023), information on environmental factors affecting species distributions on local stand scale is scarce (but see Schmitt et al. 2021). Even less studied are local‐scale distributions of sympatric tree species that hybridize. Some tree species of the genus *Quercus* (oaks) show exceptionally high rates of hybridization and introgression while partly being associated with different ecological niches (Mallet, Besansky, and Hahn 2015; Rellstab et al. 2016b). Ongoing admixture thus causes species boundaries within *Quercus* to be obscured (Gerber et al. 2014; Leroy et al. 2020; Ma, Ren, and Sun 2024). In combination with accurate microenvironmental data, oaks are therefore an ideal system to study the environmental factors driving local‐scale species distribution and admixture patterns.

The sympatric European white oak species *Quercus robur* L., *Q. petraea* (Matt.) Liebl. and *Q. pubescens* Willd. have been economically valued in Europe for centuries (Eaton et al. 2016; Haneca, Katarina, and Beeckman 2009) and have high ecological and cultural importance (Bocsi et al. 2021; Leroy, Plomion, and Kremer 2020; Mölder, Meyer, and Nagel 2019). The three species share vast parts of their distribution ranges while occupying different, but overlapping, ecological niches (Caudullo, Welk, and San‐Miguel‐Ayanz 2017; Leroy et al. 2020; Rellstab et al. 2016b). While *Q. petraea* is a drought‐tolerant generalist species, *Q. robur* tends to grow on deeper soils with higher water availability and in hydromorphic soils with stagnic or gleyic properties (Eaton et al. 2016; Rellstab et al. 2016b; Walthert and Meier 2017). The (sub‐)Mediterranean species *Q. pubescens* can tolerate extreme drought events and occurs at higher temperatures on drier soils compared to *Q. petraea* (Leroy et al. 2020; Pasta, de Rigo, and Caudullo 2016). *Quercus robur* , *Q. petraea* and *Q. pubescens* are, however, closely related, often co‐occur in mixed stands, have a substantial overlap in flowering time (e.g., Chesnoiu et al. 2009), and show moderate to high levels of genetic admixture due to recent secondary contact (Lepais and Gerber 2011; Leroy et al. 2017; Reutimann, Gugerli, and Rellstab 2020). *Quercus petraea* and *Q. pubescens* are more closely related to each other than to *Q. robur* (Leroy et al. 2017), and their degree of admixture is higher (Curtu, Gailing, and Finkeldey 2007; Lepais and Gerber 2011; Reutimann, Gugerli, and Rellstab 2020). Intraspecific gene flow is mediated through pollen dispersal, and admixture levels are highly variable between populations and individuals (Gerber et al. 2014; Reutimann et al. 2023; Reutimann, Gugerli, and Rellstab 2020). Despite ecological niche similarities and genetic admixture, the parental species remain genetically distinct and maintain their species integrity (Gailing and Curtu 2014; Reutimann, Gugerli, and Rellstab 2020).

The morphological taxonomic differentiation of the above‐mentioned oak species, especially of *Q. petraea* and *Q. pubescens*, is challenging due to an overlap of morphological characteristics and due to genetic admixture (Gugerli et al. 2007; Kremer et al. 2002; Rellstab et al. 2016a). Therefore, many morphological traits are needed for an accurate species assignment (Rellstab et al. 2016a; Viscosi et al. 2012). The morphological identification of admixed individuals is especially difficult (Rellstab et al. 2023). However, species identification and assessment of genetic admixture using molecular markers has become widely implemented (Twyford and Ennos 2012). For *Q. robur* , *Q. petraea* and *Q. pubescens* it has been shown that a few dozen of species‐discriminatory single nucleotide polymorphism (SNP) markers allow for reliable identification of the taxonomic identity of individual trees along a taxonomical gradient (Kremer et al. 2024; Reutimann, Gugerli, and Rellstab 2020).

In the *Q. petraea* /*pubescens*/*robur* species complex, it has been postulated that species co‐occurring in mixed forest stands occupy species‐specific microhabitats because of small‐scale environmental heterogeneity (e.g., Curtu, Gailing, and Finkeldey 2007; Gugerli et al. 2007). Along these lines, Reutimann et al. (2023) studied environmental and geographic factors driving admixture and species distributions of these sympatric oak species on a regional scale. They found that altitude (a proxy for temperature) and geographic position had the largest effects on the taxonomic composition of populations, while within populations, (spatially predicted) soil pH and, to a lower degree, topographic factors explained parts of the taxonomic characteristics of single trees. The authors argued that using more accurate and in situ measured environmental predictors could greatly help to assess the relevance of these findings.

Here, we investigated the environmental and geographic drivers of the distribution and admixture of the two hybridizing white oak species, *Q. pubescens* and *Q. petraea* , on local stand scale. To do so, we extensively sampled 385 trees of a single stand in Switzerland, combining genetic species assignment with high‐resolution soil measurements and topographic data. Our findings do not only contribute to the understanding of species distribution dynamics but also to the management and establishment of climate‐resilient forests.

## 2 Materials and Methods

### 2.1 Sampling Area

**FIGURE 1:**
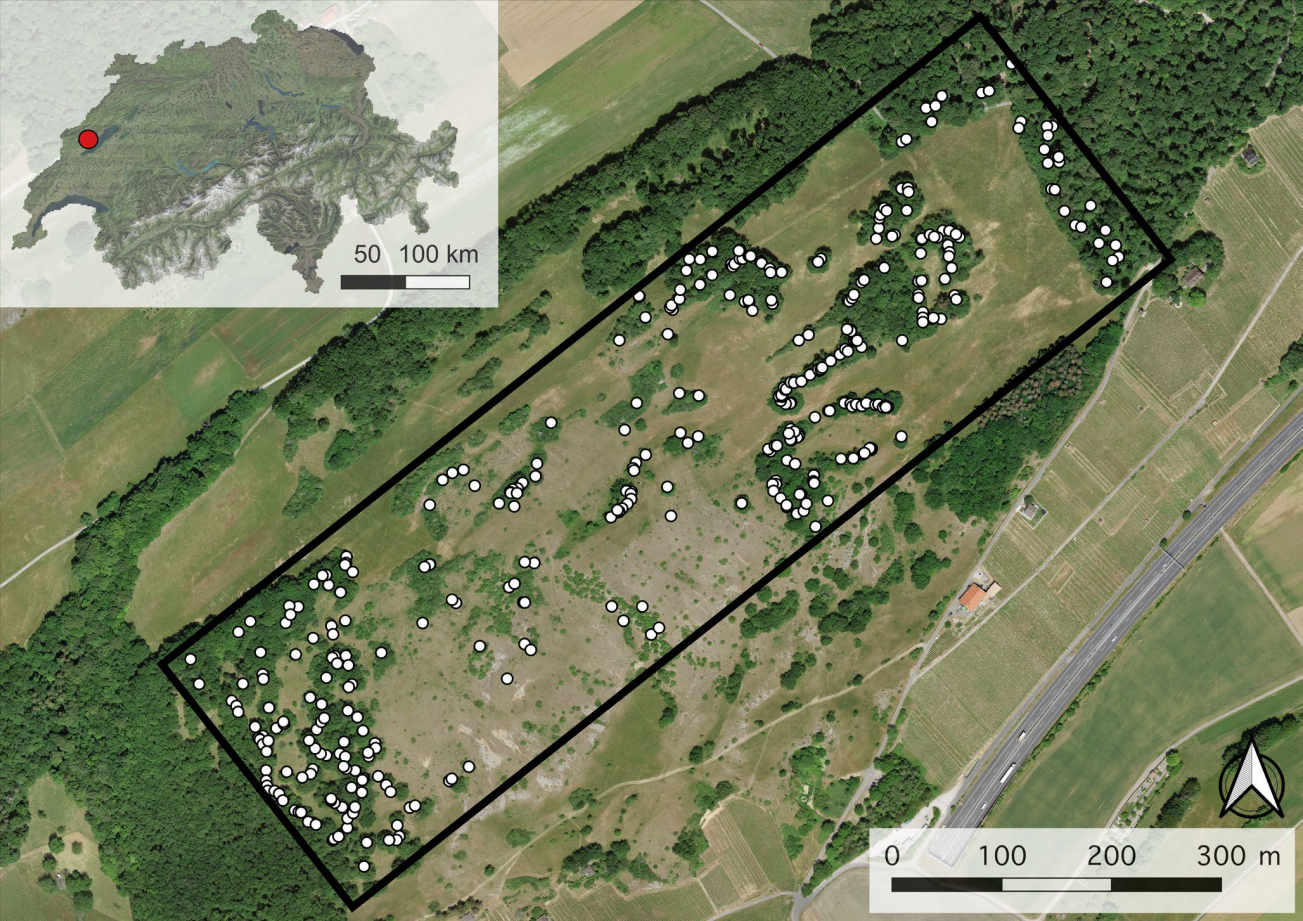
Sampling site and study design. White circles indicate the position of the 385 sampled trees. Background aerial image from Swissimage (10 cm resolution, swisstopo).

The sampling area of this study is located at the Chassagne d'Onnens on a South‐facing slope of the Jura mountains on the northwestern side of Lake Neuchâtel (Switzerland) at an elevation of around 550 m.a.s.l. (Figure 1). The Chassagne d'Onnens is a large dry meadow area with patches of naturally regenerated forest consisting of trees and shrubs. Before the 20th century, the study site was extensively used by humans, providing wood and offering acorns to feed small livestock and pigs, or foliage as supplementary fodder once the grasslands had been exhausted (personal comment D. Horisberger). During the 20th century, human activity in the area gradually declined, and today the site is only grazed annually by sheep in autumn after mechanical or manual summer maintenance. There are no records of plantings, and past human activities are unlikely to have had substantial effects on soil conditions that turned out to be relevant in the present study. Annual mean temperature ranges between 10°C and 12°C (period: 1991–2020; MeteoSwiss) with an average yearly precipitation sum of 1100–1300 mm (period: 1991–2020; MeteoSwiss). A special characteristic of the sampling site is the co‐existence of two geological substrates: glacier moraine soil in the East and limestone soil in the West (Figure S1). The fine‐scale spatial arrangements of these two substrates are characterized by a gradual transition between soil types, including mosaic‐like patterns.

### 2.2 Sampling

A total of 385 oak trees were sampled on a transect covering the different geologies found in the sampling area (950 × 275 m) (Figure 1, Figure S1). Trees were mainly selected based on diameter at breast height (DBH). For non‐isolated trees (distance to the next neighboring oak tree < 10 m), the minimum DBH was set to 15 cm. To account for a decreased tree density and to increase sampling density, the DBH threshold was reduced to 10 cm for isolated trees (distance to the next neighboring oak tree > 10 m). Such isolated trees were mainly found in the central area of the study site, where tree abundance, as well as soil depth (see Section 3), were generally low. For each tree, we recorded GPS coordinates using an Emlid Reach RS2 device (Emlid Tech Kft., Budapest, Hungary), measured DBH, and sampled leaf buds for DNA extraction, which were dried in silica gel until further processing. Additionally, we took soil samples from soil cores within 2 m of the stem (westward if possible) for every tree using a manual soil auger (3 cm diameter). For two trees standing less than 2 m apart from each other, samples from only one soil core were taken and assigned to both trees. From each soil core (maximum core depth of 130 cm [*n* = 351]), we took two soil samples for pH measurements: one topsoil sample at 0–5 cm depth (*n* = 351) and, if accessible, a deep soil sample at 40–50 cm depth (*n* = 137). Soil depth was measured by drilling a metal rod into the soil until reaching the limestone parent rock; maximum measurable soil depth was 150 cm. The depth of carbonate weathering, hereafter called lime depth, was determined by applying a 10% hydrogen‐chloride acid solution to the soil from the soil cores and visually assessing the production of carbon dioxide (Mumallah 1998). Soil samples were stored at 2°C until pH measurements.

### 2.3 DNA Extraction and Genotyping

DNA from all 385 sampled trees was extracted from 10 to 30 mg dried bud tissue using a KingFisher96 System (Thermo Fisher Scientific, Waltham, USA) with an oak‐specific sbeadex maxi plant kit (LGC Genomics, Berlin, Germany). DNA quantity and quality were analyzed with a NanoDrop spectrophotometer (Thermo Fisher Scientific, Waltham, USA) and (for a subset of the samples) with Quantus (Promega Corporation, Madison, USA). Genotyping was carried out by LGC Genomics (Hoddesdon, UK) using a Kompetitive Allele Specific PCR (KASP) assay with the best discriminating 50 out of 58 species‐discriminatory single nucleotide polymorphism (SNP) markers described in Reutimann, Gugerli, and Rellstab (2020). These SNPs were designed using mainly reference trees from Switzerland, including the Jura region, where our study site is located, but trees from other parts of Europe (Rellstab et al. 2016b). The SNPs are located in coding regions of candidate genes putatively involved in environmental adaptation (Rellstab et al. 2016b). The SNP set has been shown to reliably discriminate between *Q. robur* , *Q. petraea* and *Q. pubescens* even when the number of markers is reduced to < 30 SNPs. The selection of the 50 best discriminating markers (Table S1) was based on a threshold of a minimum 5% allele frequency difference between species pairs of the reference individuals in Reutimann, Gugerli, and Rellstab (2020). Individuals with > 10% missing data in the KASP genotyping were excluded from the dataset. Because *Q. petraea* and *Q. pubescens* are known to resprout, either naturally from roots or after coppice, there might be several trunks belonging to the same genotype in our dataset. We therefore checked our dataset for trees belonging to the same multilocus genotype using the mll function of the R package poppr 2.9.6 (Kamvar, Tabima, and Grünwald 2014) in R 4.2.2 (R Development Core Team 2022), accounting for missing data and genotyping errors using the mlg.filter function (Kamvar, Brooks, and Grünwald 2015).

### 2.4 Taxonomic Assignment

Taxonomic assignment and admixture analysis were performed as described in Reutimann, Gugerli, and Rellstab (2020). In brief, we used structure 2.3.4 (Pritchard, Stephens, and Donnelly 2000) to assign the individuals from this study based on taxonomically pure reference individuals (*N* = 194) from the study of Reutimann, Gugerli, and Rellstab (2020), which were pre‐assigned to clusters (i.e., species, *K* = 3) with the USEPOPINFO parameter implemented in structure. We ran 10 iterations and 1,000,000 repetitions after a burn‐in period of 100,000 runs. Afterwards, runs were reordered and averaged manually. When using USEPOPINFO, structure results are reported as assignment probabilities that can be interpreted analogously to ancestry coefficients (here called *Q*‐values). structure USEPOPINFO results were validated with two additional assignment approaches (Appendix S1), basic structure and the Support Vector Machine (SVM) algorithm implemented in the Rpackage e1071 1.7‐14 (Meyer et al. 2023). The Rpackage vegan 2.6.8 (Oksanen et al. 2022) was used to calculate Shannon's diversity index, an individual admixture index (*S*‐value) based on the *Q*‐values. The *S*‐value is scaled from zero (no admixture, i.e., assignment probability of 100% to one cluster) to one (individual is fully admixed, i.e., equal assignment probabilities to all clusters). All further analyses focused on *Q*‐values of *Q. petraea* and *Q. pubescens* because *Q. robur* was only present in very low frequencies. However, since genetic admixture with *Q. robur* is part of the natural hybrid system, the corresponding assignment probabilities were not excluded.

### 2.5 Soil and Topographic Data

All soil properties were derived from the in situ soil surveys. Potential rooting depth (*prd*) was calculated by averaging the soil core depth (max. 130 cm) and measured soil depth (max. 150 cm) to account for the increasing stone content towards the limestone parent rock. Maximum *prd* was thus 140 cm. Because the water storage capacity in the rooting zone of a tree is influenced by the soil's stone content, this method is supposed to be biologically more relevant than pure soil depth. Lime depth (*lid*) was assessed directly in the soil core using the hydrogen‐chloride acid method described above. In cases where no lime could be detected, *lid* was set equal to *prd* because limestone forms the bedrock for most of the sampling area. In cases where soil depth could not be determined with the methods used (i.e., because it was larger than 150 cm), it was set to 150 cm.

For every topsoil (*tph*) and deep soil (*sph*) sample, pH measurements were carried out by dissolving 20 g of fresh soil material in 0.1 M calcium dichloride (CaCl_2_) and measuring pH using a Bioblock 7.22 electrode (Fisher Bioblock Scientific, Rungis Complexe, France) (Schofield and Taylor 1955). Measurements from reference soil were used to ensure accuracy. To validate the measurements, three samples of three pH ranges of the initial measurements (pH 4.25–4.64; pH 5.6–5.82; pH 7.11–7.39) were measured five times. For spatial visualization, the soil properties were interpolated using an ordinary kriging algorithm implemented in the smart map 1.3.3 plugin (Pereira et al. 2022) in QGIS 3.28 (QGIS.org 2009).

A total of 11 topographic variables and two geographic variables (longitude [*lon*], latitude [*lat*], Table S2) were derived using a 2 m resolution digital elevation model (DEM) (swissALTI3D; vertical precision of ±0.5 m; swisstopo) based on the GPS position of individual trees. The topographic raster datasets altitude (*alt*), slope (*slp*), aspect and terrain ruggedness index (*tri*, Riley, DeGloria, and Elliot 1999) were calculated with QGIS. The remaining raster datasets were derived using SAGA 2.1.4 (Conrad et al. 2015) implemented in QGIS. The circular variable aspect was transformed into a continuous North–South (northness) gradient by using sine transformation (Guisan, Weiss, and Weiss 1999). In general, northness is more relevant in terms of insolation than eastness (East–West), because eastness only indicates slight differences in the morning and afternoon sun (Guisan, Weiss, and Weiss 1999). Therefore, only northness (*nth*) was considered in this study. For the derived topographic variables (except *slp*), a Gaussian filtering with a 6 m radius was applied to maintain the 2 m resolution while reducing noise within the data (Baltensweiler et al. 2020). Five obvious outliers were excluded from the topographic dataset.

### 2.6 Variable Selection

Initially, 17 environmental variables were analyzed in this study (four soil, 11 topographic, and two geographic variables, Table S2). The variable *sph* was excluded due to an excessive proportion of missing data (61%). Moreover, *alt* was excluded because the observed maximum altitude difference of 60 m between sampling points was not expected to have a relevant biological effect. To reduce multicollinearity of variables, pairwise Pearson's correlation coefficients (*r*) were calculated for the remaining 15 environmental variables (Figure S2). One of two highly correlated (*r* ≥ |0.75|) variables was then excluded based on its biological relevance to the study system. For example, *lat* was excluded instead of *lon* because the East–West gradient of the sampling transect is larger than the North–South gradient, and *lid* was excluded instead of *prd* because rooting depth as an indicator for water availability is expected to have a larger effect on the study system than the occurrence of carbonates. After this selection step, 11 variables remained.

To further control for multicollinearity, we computed the variance inflation factor (VIF) for each environmental variable in a linear model with *Q*‐values of *Q. pubescen*s as the response variable and the 11 explanatory environmental variables using the R package performance 0.12.3 (Lüdecke et al. 2023). Variables with a VIF > 5 and/or a VIF 95% confidence interval > 5 were excluded from the dataset (Marcoulides and Raykov 2019). Only *slp* exceeded the VIF threshold and 10 variables were kept for the analyses (Table 1).

**TABLE 1:** Final set of explanatory environmental variables after testing for multicollinearity.

Category	Abbreviation	Description	Unit	Range	References
Geographic variables	*lon*	Longitude (LV95)	m	2,541,857.23 to 2,542,703.29	—
Topographic variables	*vec*	Vertical curvature	degrees m^−1^	−0.03 to 0.03	—
	*hoc*	Horizontal curvature	degrees m^−1^	−1.51 to 2.60	—
	*ddg*	Downslope distance gradient	degrees	0.13 to 0.40	Hjerdt et al. (2004)
	*tpi*	Topographic positioning index	—	−0.38 to 0.56	Guisan, Weiss, and Weiss (1999)
	*mpi*	Morphometric protection index	—	0.04 to 0.15	Yokoyama, Shirasawa, and Pike (2002)
	*twi*	Topographic wetness index	—	2.48 to 8.49	Böhner et al. (2002)
	*pti*	Potential total insolation	kWh m^−2^	5,425,326.00 to 6,453,819.50	Böhner and Antonić (2009)
Soil variables	*prd*	Potential rooting depth	cm	4 to 140	—
	*tph*	Topsoil pH (0–5 cm)	—	3.39 to 7.69	—

### 2.7 Identifying Environmental Factors Driving Taxonomic Structure and Admixture

All analyses in this section were carried out with assignment probabilities (*Q*‐values) or admixture indices (*S*‐values, except for the partial redundancy analysis [pRDA]) as response variables and environmental variables (*E*‐values, Table 1) as explanatory variables. Except for the pRDA, only *Q*‐values of *Q. pubescens* were used because *Q*‐values of *Q. pubescens* and *Q. petraea* were nearly reciprocal due to the low abundance of *Q. robur* (see Section 3). Additionally, a reduced dataset (*n* = 237 trees) based on a narrower sampling transect (Figure S3) was analyzed to control for the effects of environmental extremes that were found at both ends of the sampling transect and to narrow down the analyses to the center of the sampling site where environmental conditions are more variable and mosaic‐like. All *E*‐values were centered (average = 0) and scaled (standard deviation [SD] = 1). When *S*‐values were used as response variable, the analyses were also performed with squared *E*‐values to investigate whether intermediate *E‐*values exhibit higher admixture levels.

#### 2.7.1 Generalized Linear Models

Generalized linear models (GLMs) with a stepwise selection (forward and backward) were used as described in Reutimann et al. (2023) to identify the key factors influencing species distribution and admixture levels within the stand. GLMs are particularly suitable in this context because they accommodate non‐normal response variables and allow the quantification of effect sizes. The gamlss function of the R package gamlss 5.4‐12 (Stasinopoulos et al. 2023) was used to create the following GLMs: *Q*‐values ~ *E*‐values; *S*‐values ~ *E*‐values; *S*‐values ~ (*E*‐values)^2^. GLMs allow the linear modeling of non‐normally distributed response variables using specific link functions (Nelder and Wedderburn 1972). A logit‐normal (LOGITNO) distribution family was chosen to model the response variables (*Q*‐values), which are bound between zero and one. Alternatively, a beta‐distribution family is proposed to be used when dealing with proportional response variables (Douma and Weedon 2019). However, the highly similar LOGITNO distribution had a better fit for the models used based on a Q‐Q plot of the full transect analysis (Figure S4). For the normally distributed *S*‐values, a normal distribution family was used. After running the GLM selection with default parameters (forward and backward), the models with the highest explanatory power were selected based on Akaike information criterion (AIC) using the stepgaic function of the gamlss package in R.

#### 2.7.2 Bayesian Model Averaging

In addition to the GLMs, a Bayesian model averaging (BMA) was performed with the R package bms 0.3.5 (Hofmarcher, Stefan, and Paul 2022) as described in Reutimann et al. (2023). In contrast to GLMs (which result in the single best‐predicting model), BMA indicates which explanatory variables are included in how many of the n most accurate models (Fragoso, Bertoli, and Louzada 2018) and therefore more widely explores the model parameter space than the single GLMs. The same linear models as in the stepwise model selection procedure were used for BMA. The bms function uses Markov Chain Monte Carlo (MCMC) to infer posterior inclusion probabilities of different explanatory variables, posterior model probabilities, as well as posterior model sizes. The BMA was run with 3,000,000 iterations (1,000,000 burn‐in) for the best 1000 models with a uniform model prior. All other settings were kept on default.

#### 2.7.3 Partial Redundancy Analysis

To assess the effect of *E*‐values on taxon assignment probability in a multidimensional space and to account for the potential effect of geographic position (*lon*) on the *Q*‐values, a pRDA was carried out using the R package vegan. Here, the *Q*‐values of all three species were used since this method allows the use of multiple response variables. All ten uncorrelated environmental variables (Table 1) were used as explanatory variables. The pRDA allows for control of confounding effects by partialling out the effect of explanatory variables, in this case *lon*, on the response variables before performing a standard RDA (Buttigieg and Ramette 2014). This allows removing intrinsic (spatial) effects of the conditional variables on the relationship of taxonomic structure and environment (Borcard, Legendre, and Drapeau 1992). Analysis of variance (ANOVA) was used to validate the significance of the pRDA model and axes. Results were visualized in an ordination plot using the ordiplot function implemented in vegan. To further investigate small‐scale patterns of taxonomy and environment, we assessed the degree of spatial autocorrelation. We calculated Mantel correlograms testing the effect of the geographical distance among trees on the distances of genotypes (based on taxonomic proportion of *Q. pubescens*) and of the two most important environmental descriptors (see Section 3) with the R package vegan.

### 2.8 Ecological Niche of Admixed Individuals

To have a closer look at the specific habitats (regarding only the most important environmental descriptors, see Section 3) of pure and admixed individuals, the sampled trees were categorized into pure *Q. petraea* (*Q*‐value < 0.1), pure *Q. pubescens* (*Q*‐value > 0.9), and admixed individuals (*Q*‐value between 0.1 and 0.9). To statistically infer significant differences between these groups, environmental data were transformed with a Box‐Cox transformation, followed by an ANOVA and a Tukey's post hoc test using R.

## 3 Results

### 3.1 Genotyping and Taxonomic Assignment

Five of 385 genotyped trees were excluded from the taxonomic assignment due to a large proportion of missing data (> 10%). In total, 21 trees shared their multilocus genotype with at least one other tree. Identical multilocus genotypes were located between 1.3 m and 219.5 m apart. Only two pairs of trees exhibited a distance of less than 5 m, whereas the vast majority of the trees sharing the same multilocus genotype were over 100 m apart. We therefore did not exclude identical multilocus genotypes, because closely located trees did not show an enrichment for identical multilocus genotypes, and the marker set used in the study was designed to be species‐diagnostic and might not necessarily identify individual genotypes (Reutimann, Gugerli, and Rellstab 2020).

**FIGURE 2:**
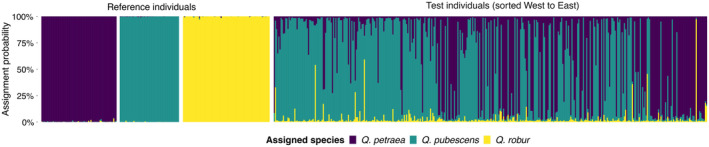
Taxonomic assignment of the sampled trees. structure plot showing assignment probabilities for reference and test individuals to the three species *Quercus petraea* , *Q. pubescens*, and *Q. robur* . Samples were analyzed with 50 species‐diagnostic single‐nucleotide polymorphism (SNP) markers. Each bar represents a single tree, and the colors represent assignment probabilities for the respective species. Reference individuals (left, *N* = 194) are grouped by species. Test individuals (right, *N* = 380) are sorted from West to East.

*Q*‐values of the structure analysis with *K* = 3 using the USEPOPINFO parameter (Figure 2) were confirmed by two alternative taxonomic assignment methods, basic structure (linear regression of *Q. pubescens Q*‐values, *R* ^2^ = 1, *p* < 0.001) and SVM (*R* ^2^ = 0.91, *p* < 0.001, Appendix S1). The choice of *K* = 3 was confirmed by the likelihood of *K* of the basic structure analysis (Figure S5). The basic structure analyses for *K* = 2–5 (Figure S6) show that *K* = 2 splits *Q. robur* from the rest of the samples, whereas all three species are clearly separated with *K* = 3. Increasing *K* beyond three results in further clusters within *Q. pubescens* and *Q. petraea* , as visible in the reference individuals (Figure S6).

The mean *Q*‐value of the sampled trees for *Q. pubescens* was 0.57 (median = 0.80), for *Q. petraea* 0.40 (median = 0.16), and for *Q. robur* 0.03 (median = 0.01). *S*‐values (admixture index) ranged from 0.11 to 0.99 with an average of 0.33 (median = 0.26, Figure S7). Using a *Q*‐value threshold of 0.9 for classifying a tree as a pure individual, 145 *Q. pubescens*, 104 *Q. petraea* , one *Q. robur* , and 130 admixed trees were identified among the test individuals.

### 3.2 Environmental Data and Variable Selection

The accuracy of the pH measurements was high (average coefficient of variation = 2.41%, average SD = 0.13). The in situ measured soil variables potential rooting depth (*prd*, Figure S8), lime depth (*lid*, Figure S9), and topsoil pH (*tph*, Figure 10) were spatially interpolated from 385 measurements, whereas deep soil pH (*sph*, Figure S11) was interpolated from 156 measurements. The inferred soil patterns (Figures S8–S11) corresponded well to the available geological information (Figure S1). On the eastern side of the sampling area with the glacier moraine geology, the soil is deeper, with a deeper lime depth and a lower soil pH. On the western side, with the limestone geology, the soil is shallower, with a shallower lime depth and a higher soil pH. However, in comparison to the geological map (Figure S1), soil conditions were more mosaic‐like, especially in the center of the sampling transect, and often intermediate compared to the rather uniform and extreme conditions in the western and eastern parts of the study area. Therefore, in addition to the whole transect, using a reduced transect that excluded the western and eastern extremes allowed a more fine‐scaled analysis of the influence of soil properties on the taxonomic structure.

### 3.3 Detection of Environmental Factors Driving Taxonomic Structure and Admixture

**FIGURE 3:**
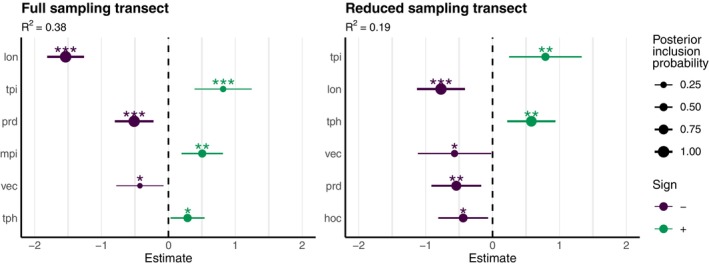
Environmental variables with a significant effect on the taxonomic proportion of *Quercus pubescens* in individual trees. Significant environmental variables (see Table 1 for details) and their coefficients resulting from generalized linear models with assignment probabilities (*Q*‐values) of *Q. pubescens* as the response variable. Results are shown for the full (left figure) and reduced (right figure) sampling transects. The horizontal bars indicate the 95% confidence intervals as estimated from the models. *R* ^2^‐values indicate the explanatory power of each model. Significance levels are indicated as follows: *p < 0.05; **p < 0.01; ***p < 0.001. Points are sized according to the posterior model inclusion probability of each variable and colored according to their sign inferred from the Bayesian model averaging.

The best‐explaining GLM (AIC = −527.14), as identified by the model selection procedure for the full transect dataset, explained 38% of the variation of *Q. pubescens Q*‐values. The model included, in order of decreasing effect (Figure 3), the significant variables longitude (*lon*), topographic positioning index (*tpi*), *prd*, morphometric protection index (*mpi*), vertical curvature (*vec*), and *tph*. Taxonomically, more *Q. pubescens* and less *Q. petraea* ‐like trees were thus predicted to be located more in the West, on shallower soil, in locations with lower *vec*, higher *tpi*, higher *mpi*, and higher *tph*. The BMA mostly confirmed these results, where *lon* (posterior inclusion probability [PIP] = 1.00) and *prd* (PIP = 0.99), followed by *tph* (PIP = 0.53), showed high PIPs (Figure 3, Figure S12). The best predicting model consisted of these three parameters, and, therefore, the indicated importance of these variables resulting from the GLM is strongly supported by BMA. Additionally, *mpi* (PIP = 0.50) was strongly supported by the BMA.

When the reduced transect dataset was modeled in a GLM, the results were similar, but the spatial effect of *lon* was weaker while the model had less explanatory power (*R* ^2^ = 0.19, Figure 3). The best explaining model (AIC = −275.58) included, in order of decreasing effect, *lon*, *tpi*, *tph*, *vec*, *prd*, and *hoc*. The sign of the coefficients for the effects was the same as in the full transect GLM. Importantly, the effect size of *lon* (estimate = −1.54) was about 3x higher than the one of *prd* (estimate = −0.51) in the full transect GLM, whereas in the reduced transect GLM, the effect size of *lon* (estimate = −0.77) was only about 1.5x higher than the one of *prd* (estimate = −0.54). Compared to the full transect GLM, the topographic variable *hoc* instead of *mpi* had a significant negative effect on the *Q. pubescens Q*‐values. Similar to the full model, the BMA approach confirmed the sign and importance, in decreasing order, of the variables *lon* (PIP = 0.98), *tph* (PIP = 0.88), *prd* (PIP = 0.77), and *hoc* (PIP = 0.60) (Figure 3, Figure S12). These four variables were also included in the model with the highest probability.

The effect of unsquared or squared environmental variables on admixture levels (*S*‐values) of the trees was negligible in both the full and reduced transects. Although some topographic variables were significant in the best‐explaining GLMs, these models had very low explanatory power (*R* ^2^ full transect = 0.02; *R* ^2^ reduced transect = 0.02), and none of the variables were included in the best‐explaining model of the BMA.

**FIGURE 4:**
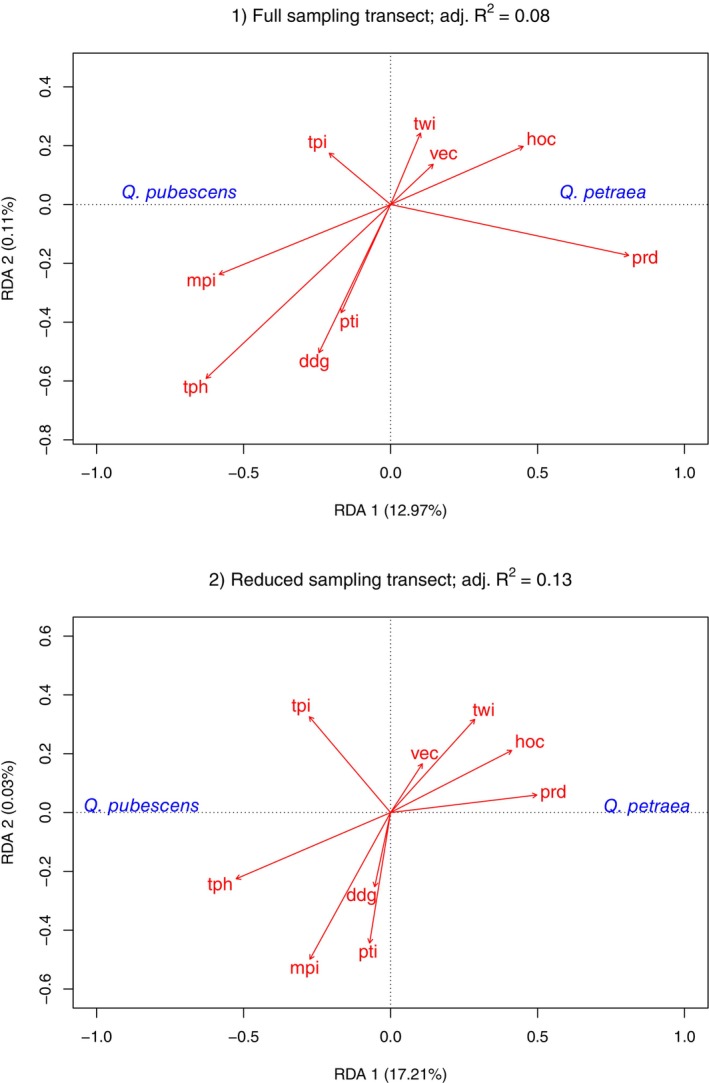
Partial redundancy analysis showing the contribution of environmental variables to the taxonomic variation of *Quercus* spp. while accounting for the effect of geographic position. Species are indicated in blue, and environmental variables (see Table 1 for details) in red. Arrows display the direction of effects of environmental variables on taxonomic variation. Arrow length shows the strength of the effect. Adjusted *R* ^2^‐values indicate the explanatory power of the model. The constrained variation explained by axis 1 (RDA1) and axis 2 (RDA2) is indicated in brackets.

In the pRDA for the full transect dataset (*p* < 0.001, adj. *R* ^2^ = 0.08), the conditional variable *lon* contributed 28.84% of the variance to the trees' taxonomic variation (Figure 4). Another 9.31% of the variation was explained by the environmental variables, whereas 61.86% of the variation remained unexplained. The first canonical axis of the pRDA was significant (*p* < 0.001) and confirmed the results of the GLMs and BMA because *prd* (0.67) and *tph* (−0.52) had the highest biplot scores. In the pRDA of the reduced sampling transect (*p* < 0.001, adj. *R* ^2^ = 0.13, Figure 4), *lon* explained a total of 4.25% of variance in tree taxonomy. The other environmental variables explained 16.51%, and 79.24% remained unexplained. The two most relevant explanatory variables in this analysis were again *prd* and *tph*, with biplot scores of 0.55 and −0.58 on the first RDA axis. In summary, the analysis of the reduced transect exhibited a higher explanatory power, a smaller effect of geographic position, and more variance explained by soil variables compared to the full transect.

**FIGURE 5:**
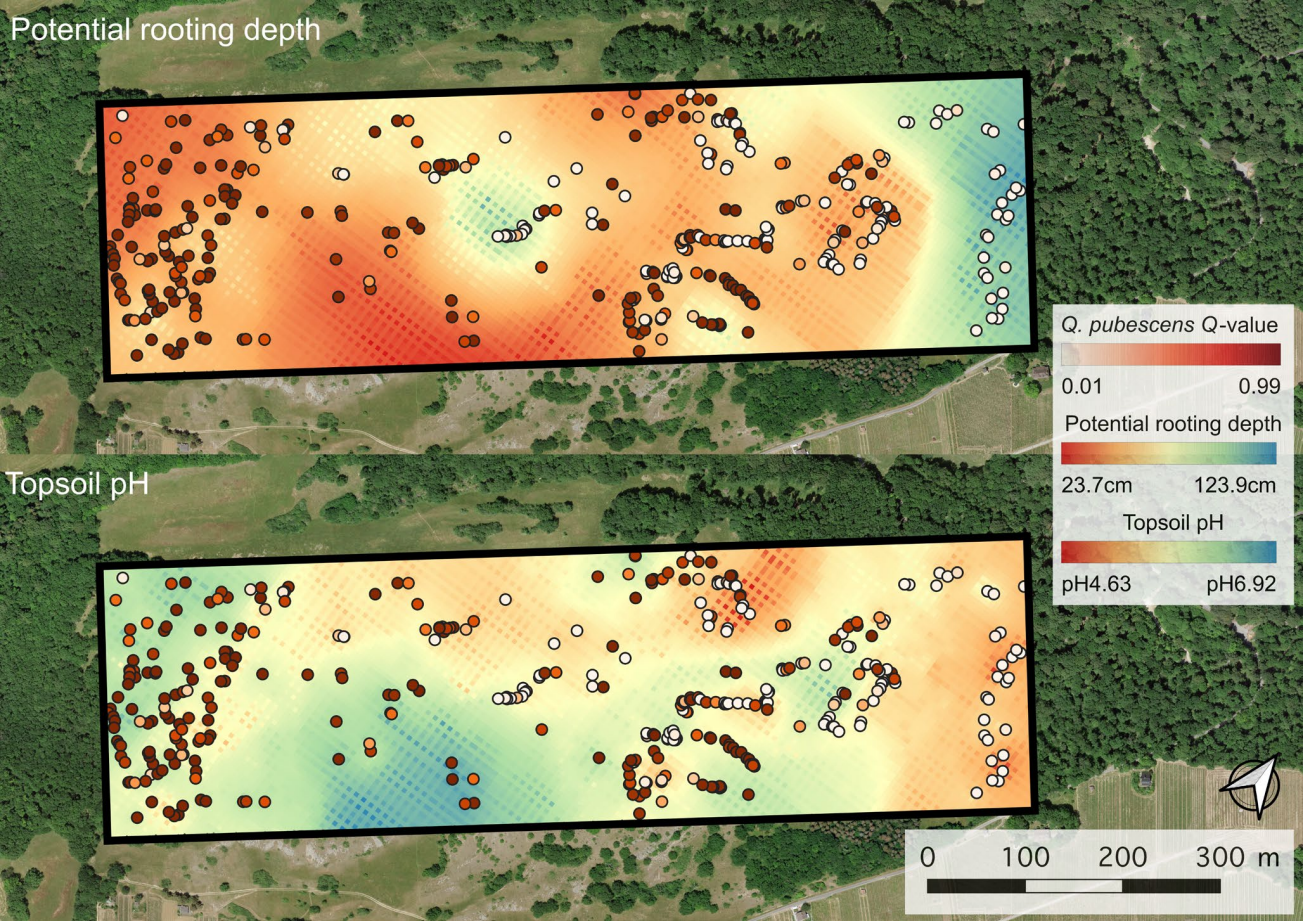
Tree taxonomy on interpolated soil maps. Two maps displaying potential rooting depth (top) and soil pH (bottom) derived and interpolated from in situ measurements. Individual trees (circles) are colored according to *Quercus pubescens Q*‐values. Background aerial image from Swissimage (10 cm resolution, swisstopo).

In summary, besides *lon*, *tph* and *prd* were consistently among the best variables in the GLMs, BMA, and pRDA, explaining a substantial part of the taxonomic composition of the sampled trees. All analyses showed that trees with a high taxonomic proportion of *Q. pubescens* can be found in locations with higher soil pH and shallower potential rooting depth, whereas *Q. petraea* shows the opposite pattern. This can also be observed in the prediction of *Q. pubescens Q*‐values using *tph* and *prd* as explanatory variables (Appendix S2). Importantly, these patterns could not only be observed across the entire site from East to West, but also within smaller‐scale, mosaic‐like patches of the oak stand (Figure 5). For example, the *Q*‐values of trees in the island‐like area with a large *prd* in the middle of the sampling transect were less *Q. pubescens* and more *Q. petraea‐like*. Such fine‐scale patterns were even more evident for *tph*, with several islands of high or low pH occupied by the respective tree species. The topographic variables did not show such explicit spatial patterns of correlation to the genetic data (Figures S13–S19). Moreover, these fine‐scale patterns were confirmed by analysis of spatial autocorrelation (Figure S20); there was a rather weak (max. correlation coefficient 0.1), but significant autocorrelation for genotype (up to 300 m), followed by *prd* (up to 150 m) and *tph* (below 100 m). Autocorrelation for *prd* and genotype was significant again at a distance of around 400–500 m, likely related to the island‐like patterns visible in Figure 5.

### 3.4 Ecological Niche of Admixed Individuals

**FIGURE 6:**
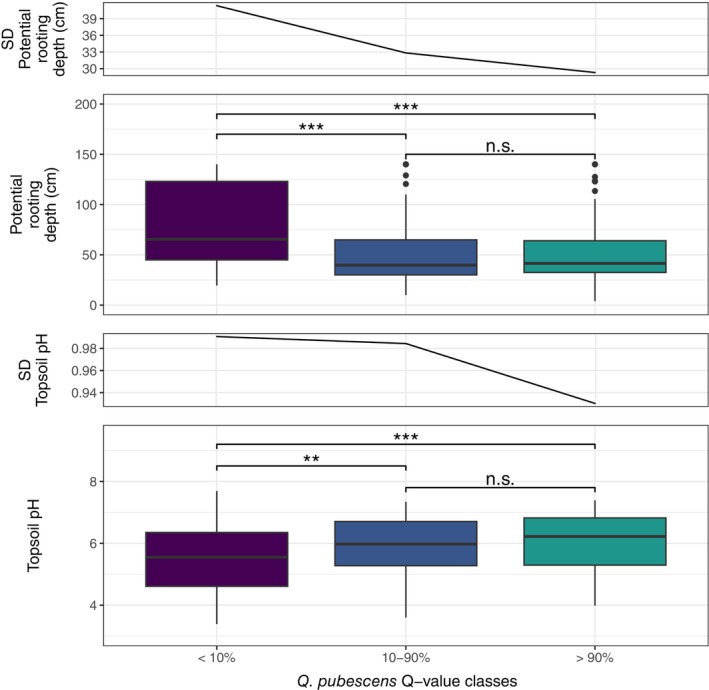
Soil properties of pure and admixed individuals. Boxplots showing the distribution of potential rooting depth (top) and topsoil pH (bottom) grouped by taxonomic categories (pure *Quercus petraea* : *Q*‐value < 10%; admixed individuals: *Q*‐value = 10%–90%; pure *Q. pubescens*: *Q*‐value > 90%). Line plots on top of the boxplots indicate the variation (measured as standard deviation) for potential rooting depth (top) and topsoil pH (bottom). Significance levels of the Tukey's post hoc test between groups are indicated above the boxplots as follows: ^n.s.^ *p* > 0.05; *p < 0.05; **p < 0.01; ***p < 0.001.

Pure *Q. petraea* individuals were found in locations with the largest average environmental variability (SD) of *prd* and *tph* (Figure 6). Pure *Q. pubescens* trees were found in locations with the lowest average SD, whereas admixed individuals were found in locations with intermediate levels of SD of these environmental variables. Furthermore, the three taxonomic groups were significantly different based on the ANOVA regarding both environmental variables, *prd* (*p* < 0.001) and *tph* (*p* < 0.001). Based on post hoc testing, only pure *Q. petraea* individuals were found in significantly different conditions than the other two taxonomic groups (adj. *p* < 0.01).

## 4 Discussion

Knowledge about environmental and geographic factors driving the distribution and admixture of trees and other plant species on a local scale can be key for the establishment, management, and persistence of resilient forests and ecosystems in the future. Because intraspecific genetic variability may not be sufficient to adapt to a rapidly changing climate, interspecific hybridization could potentially accelerate these adaptation processes through the introgression of adaptive alleles (Suarez‐Gonzalez, Lexer, and Cronk 2018; Wong, Hiscock, and Filatov 2022). In this study, we genotyped 385 white oaks (mainly *Q. petraea* and *Q. pubescens*) in a single stand using species‐diagnostic SNP markers and tested how the taxonomic composition and admixture levels correlate with geographic, topographic, and in situ measured soil properties on a local scale. We could show that the taxonomic composition is mainly explained by geographic position and micro‐edaphic conditions (potential rooting depth and topsoil pH). Furthermore, we found that admixed individuals of the sampled oak population were not found in intermediate environmental conditions but rather in those that are similar to the habitats of *Q. pubescens*.

### 4.1 Model Performance and Comparison

The Bayesian model averaging (BMA) approach can be seen as an addition to the generalized linear models (GLMs) as it provides further information on variable inclusion and model probabilities. The variables included in the ten best models in the BMA were also included in the GLMs as significant variables, with the most significant variables showing the highest inclusion probabilities in BMA. The explanatory power of the GLMs was moderate (*R* ^2^ full transect = 0.38; *R* ^2^ reduced transect = 0.19), indicating that a part of variation in the taxonomic structure remained unexplained. Also, the explanatory power of the partial redundancy analyses (pRDAs) in this study was rather low (adj. *R* ^2^ full transect = 0.08; adj. *R* ^2^ reduced transect = 0.13), but the approach allowed us to analyze the effects of environmental variables while conditioning on geographic position. A reason for the moderate explanatory power could be that the derived variables do not describe all biologically relevant abiotic differences and/or that there is some stochasticity involved in the establishment of the trees. Furthermore, it is likely that unmeasured biotic factors like competition, seed and pollen dispersal, as well as human influences, affect taxonomic composition. Especially pollen dispersal has been shown to be a major driver of genetic admixture in sympatric oak populations (Gerber et al. 2014). However, the fact that all three approaches (i.e., GLMs, BMA, and pRDAs) identified geographic position, potential rooting depth, and topsoil pH as important variables indicated that we could pinpoint some of the main factors related to the local scale taxonomic distribution in this white oak stand.

### 4.2 Biological Drivers of Taxonomic Structure

Of all variables tested, longitude had the strongest effect on the taxonomy of the trees in both the GLMs and the BMA. Trees that were located towards the East consisted taxonomically of less *Q. pubescens* and more of *Q. petraea* . Since longitude was moderately correlated to many other environmental variables (Figure S2), it seems likely that the effect is either caused by an East–West gradient in other measured environmental variables or that there is such a gradient in unmeasured factors like, e.g., wind direction affecting pollen dispersal, abundance of animal seed dispersers, or competition (e.g., Gerber et al. 2014). Another potential explanation for the strong East–West pattern might be the history of the study site, e.g., that two sub‐populations composed of two different species came into contact after deforestation. To limit the strong longitudinal effect, we ran all analyses on a reduced dataset that included only the central part of the study site where environmental conditions were much more mosaic‐like rather than East–West oriented. The reduced transect models showed less explanatory power but increased the relative effect of the soil variables in comparison to longitude. This indicates that longitude might actually mask a part of the effects of the soil variables.

The two environmental variables that had a consistent and strong effect on the taxonomic structure were potential rooting depth and topsoil pH. Trees found on shallower and/or more alkaline soils were composed of a higher *Q. pubescens* and lower *Q. petraea* genomic proportions. Potential rooting depth can be seen as a proxy for water availability (Stocker et al. 2023). It has been shown in various studies on larger scale that *Q. pubescens* is associated with drier soils than *Q. petraea* (Rellstab et al. 2016b; Walthert and Meier 2017), indicating species‐specific differences in drought resistance (but see Arend et al. 2011). This might come from differences in e.g., vulnerability to air embolism (Cochard et al. 1992), water loss after stomatal closure, risk of soil‐root capillary disruption, or dehydration tolerance of parenchyma and meristem cells (discussed in Körner 2019). Similarly, it has been shown before that *Q. pubescens* can often be found on soils with higher pH than *Q. petraea* (Leroy et al. 2020; Rellstab et al. 2016b; Reutimann et al. 2023). Differences in soil pH might be related to the availability of nutrients (Härdtle et al. 2004); however, it has been shown that nutrients play only a minor role in explaining the distribution of the two species at large scale (Walthert and Meier 2017). Moreover, *Q. pubescens* can also be found on crystalline bedrock with low pH (Rellstab et al. 2016b). Therefore, soil pH might not have a direct impact on the occurrence of the different taxa but could rather represent an additional variable describing the two soil types and their transition.

In addition to these soil variables, several topographic variables were found to potentially influence the taxonomic structure of the investigated forest stand. For example, the topographic positioning index (i.e., exposure; De Reu et al. 2013) and morphometric protection index (i.e., protectedness by the surrounding topography, Yokoyama, Shirasawa, and Pike 2002) were associated with a higher genomic composition of *Q. pubescens*. Furthermore, trees that were found in more convex locations (larger vertical and horizontal curvature) were genetically more *Q. petraea* and less *Q. pubescens* like. In the case of topographic positioning index and horizontal curvature, this confirms the within‐population findings and even effect signs of Reutimann et al. (2023) based on a smaller number of trees. However, we consider the biological importance of these variables in our study system relatively small. First, the inconsistency among the different statistical analyses weakens the plausibility of these variables having a strong causal effect. Second, the topographic variation along the entire transect was rather small (Figure S3), leading to a low range in some of these variables. For example, the range in topographic positioning index (0.94) was substantially smaller than in e.g., Reutimann et al. (2023) (0.86–11.34 within populations). Investigations in additional, topographically more variable populations are therefore needed to confirm the findings of our models.

The two most important environmental characteristics studied here—water availability and pH—therefore not only seem to be strongly associated with the distribution of the two species on a large (Rellstab et al. 2016b) and regional scale (in the case of pH; Reutimann et al. 2023) but also on a small local spatial scale within a single site. Such a strong effect of soil properties on tree taxonomy has, to our knowledge, never been directly shown before on such a small scale. The interpolated soil maps (Figure 5) illustrate how the patchiness in potential rooting depth and topsoil pH is in concordance with the fine‐scale taxonomic distribution of the two species and their admixed individuals. For example, the island‐like, 50–100 m wide region with higher soil depth in the center of the site coincides with (almost) pure *Q. petraea* trees, while the taxonomy of the trees around that region is more variable. Additionally, spatial autocorrelation of genotypes and soil variables quickly decreased with increasing distance (and increased again at larger distances), also pointing to small‐scale and island‐like patterns (Figure S20). Similarly, Truffaut et al. (2017) found small‐scale environmental differences associated with taxonomic differences in a mixed stand of *Q. petraea* and *Q. robur* showing signs of genetic admixture. In that study, soil properties were not directly measured in the field but inferred and interpolated from ecological indicator values. In summary, our and the previously mentioned study suggest that the spatial distribution of hybridizing oak taxa in a mixed stand is to a large degree determined by environmental differences in local soil conditions, especially water availability.

### 4.3 Ecological Niches of Admixed and Pure Individuals

The hybrid‐superiority hypothesis assumes that hybrid fitness is enhanced in intermediate environments compared to the fitness of the parental species (Moore 1977). Admixed individuals are thus often found to be morphologically and ecologically intermediate (Kamiński et al. 2020; McDade 1990). Here, admixed individuals were found in habitats similar to the parental species *Q. pubescens*. Admixed individuals were also not found in locations with a wider variation in environmental factors (i.e., wider niche) than in those of pure individuals. Our results support previous studies showing that *Q. petraea* is a generalist species (Rellstab et al. 2016b); here the species was found in locations with a wide range of potential rooting depth as well as topsoil pH. Because *Q. pubescens* is a more drought‐resistant species, admixture of *Q. petraea* with *Q. pubescens* could be largely beneficial for increasing drought resistance in *Q. petraea* populations. The above‐described study of Truffaut et al. (2017) investigated the progression of taxonomic structure in a sympatric *Q. petraea* and *Q. robur* population and found that, under drought stress, *Q. petraea* can make better use of the limited available water resources than *Q. robur* . In conclusion, *Q. robur* populations admixed with *Q. petraea* may have a selective advantage over pure *Q. robur* populations under dry conditions (Truffaut et al. 2017). Similarly, *Q. petraea* populations admixed with *Q. pubescens* may have an advantage over pure *Q. petraea* populations (this study). Introgression therefore represents a possible mechanism to cope with rapidly changing environmental conditions.

## 5 Conclusions

The results of this study indicate that the distribution of *Q. petraea* , *Q. pubescens* and their admixed individuals at a very small spatial scale is not random but at least partially driven by local soil properties, in particular soil water availability and pH. Diversity in these microenvironmental factors can therefore promote taxonomic diversity in oak populations which may allow oak stands in environmentally diverse locations to better adapt to environmental changes through adaptive introgression (Lazic et al. 2021; Leroy et al. 2020). However, it is important to acknowledge that our findings are based on a single stand. Generalization of these results to other species, populations or environmental contexts needs further studies across multiple species pairs, stands, or regions. Our study also emphasizes the need for in situ measurements of environmental and biological descriptors to get a more detailed picture of the environmental drivers of species distributions. Including information about intraspecific taxonomic variation could further increase the precision of a species' predicted range under future climatic conditions. The fact that genetically admixed individuals between *Q. pubescens and Q. petraea* were predominantly found in habitats similar to *Q. pubescens* implies an interesting potential for forest management for increasing the drought resistance in *Q. petraea* populations by allowing or promoting admixture with *Q. pubescens*. For example, in locations with currently suitable environmental conditions for both species, *Q. petraea* populations could be enriched with *Q. pubescens* gene variants to achieve such a goal.

## Conflicts of Interest

The authors declare no conflicts of interest.

## Supporting information


Data S1.

## Data Availability

Raw data (individual genotypes, taxon assignments, environmental/geographic data and DBH data) is available on the Dryad Digital Repository (DOI: 10.5061/dryad.n8pk0p35w). The complete collection of input files, R‐scripts, and output files (including structure) can be found on https://gitlabext.wsl.ch/felix.zimmermann/2025_eva_quercus_taxonomic_diversity. In this study, the Nagoya Protocol is not applicable and there are no benefits to report.
