# Supplementary material for: Fine‐Scale Variation in Soil Properties Promotes Local Taxonomic Diversity of Hybridizing Oak Species (Quercus spp.)
Source: Evol Appl. 2025 Feb 6;18(2):e70076. doi: 10.1111/eva.70076 (PMC11802334; doi:10.1111/eva.70076)
Supplement: Supplementary file 1 — Data S1. [file EVA-18-e70076-s001.docx]

**Supplementary materials for the article:**

**Fine-scale variation in soil properties promotes local taxonomic diversity**

**of hybridizing oak species (*Quercus* spp.)**

**Authors**

*Felix Zimmermann*^1^ (felix.zimmermann@wsl.ch; ORCID: 0009-0002-0762-2454),

*Oliver Reutimann*^2^ (oliver.reutimann@usys.ethz.ch; ORCID: 0000-0002-5042-7518),

*Andri Baltensweiler*^1^ (andri.baltensweiler@wsl.ch; ORCID: 0000-0003-1933-6535),

*Lorenz Walthert*^1^ (lorenz.walthert@wsl.ch; ORCID: 0000-0002-1790-8563),

*Jill K. Olofsson*^3^ (jko@ign.ku.dk; ORCID: 0000-0002-9527-6573) &

*Christian Rellstab*^1^* (christian.rellstab@wsl.ch; ORCID: 0000-0002-0221-5975)

^1^ Swiss Federal Research Institute WSL, Birmensdorf, Switzerland

^2^ Institute of Integrative Biology, ETH Zurich, Zurich, Switzerland

^3^ Section for Forest and Landscape Ecology, Department of Geosciences and Natural Resource Management, University of Copenhagen, Denmark

* Corresponding author: christian.rellstab@wsl.ch

***Supplementary Figure 1: Sampling area on geological atlas background map.***

*White circles indicate the position of the 385 sampled trees in this study. Colors indicate different soil types. Source: Geological Atlas of Switzerland 1:25,000; ©swisstopo.*


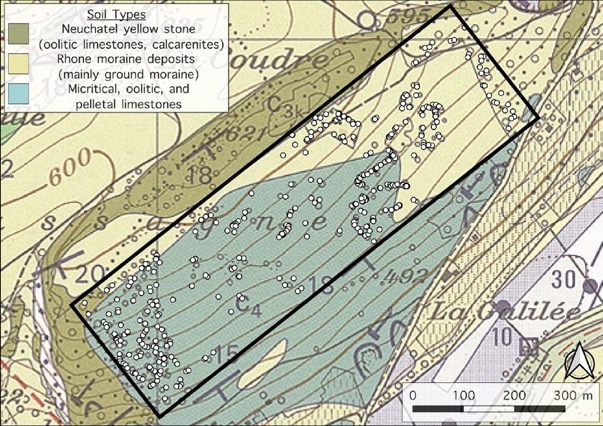


***Supplementary Figure 2: Pairwise Pearson’s correlation coefficients (r) for all environmental variables.***

*Negatively correlated variables included in the models are colored in red and positively correlated variables are colored in blue. Color intensity indicates the strength of correlation. For details and abbreviations see Tables 1 and Suppl. Table 2.*

**

***Supplementary Figure 3: Illustration of the full and reduced sampling transects, sampling points and topography of the sampling area.***

*Black: Full dataset (n=385), red: reduced dataset (n=237). Source: swissALTI3D, ©swisstopo.*

***
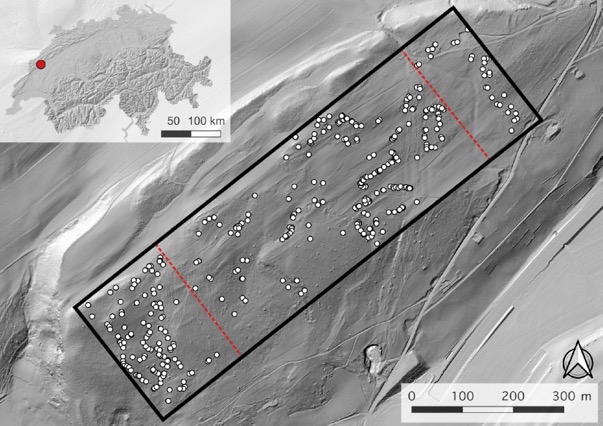
***

***Supplementary Figure 4: Q-Q-plot of residuals resulting from GLMs with different distribution families.***

*Residuals of beta-distribution model (BE) are colored in red and residuals of logit-normal distribution model (LOGITNO) are colored in blue.*

**

***Supplementary Figure 5: Likelihood of K in the basic Structure*** ***analysis.***

*Mean estimated logarithmic probability L(K) for the number of K clusters over 10 repeated runs.*

******

***Supplementary Figure 6: Basic Structure*** ***results for K = 2 – 5 clusters.***

*Each bar represents a single tree. The colors represent assignment probabilities to respective clusters (i.e. species in K=3). Reference individuals (N = 194) are grouped by species on the left side of the figure. Test individuals (N = 380) are sorted from West to East and presented on the right side of the figure.*

******

***Supplementary Figure 7: Admixture levels of test individuals.***

*Test individuals (N = 380) are sorted from low to high (left to right) admixture levels (S-values, red dots). The colors denote assignment probabilities to respective clusters based on Structure with USEOPOPINFO.*

******

***Supplementary Figure 8: Interpolated potential rooting depth (prd).*** *Sampling points are shown as circles on top of the interpolated map and colored according to field measurements. Background map: SWISSIMAGE 10 cm; ©swisstopo.*

*
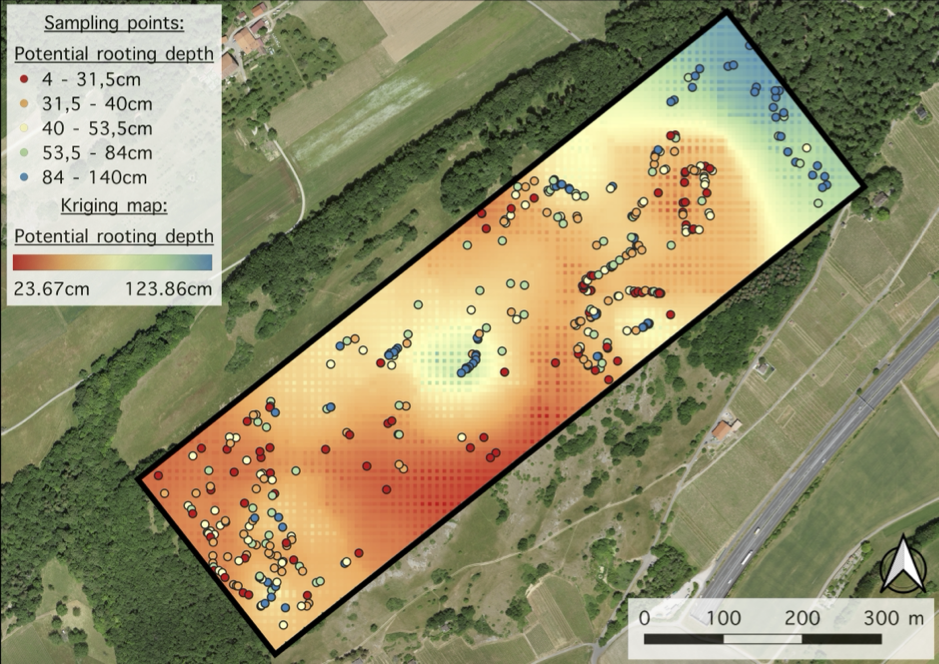
*

***Supplementary Figure 9: Interpolated lime depth (lid).*** *Sampling points are shown as circles on top of the interpolated map and colored according to field measurements. Background map: SWISSIMAGE 10 cm; ©swisstopo.*

***
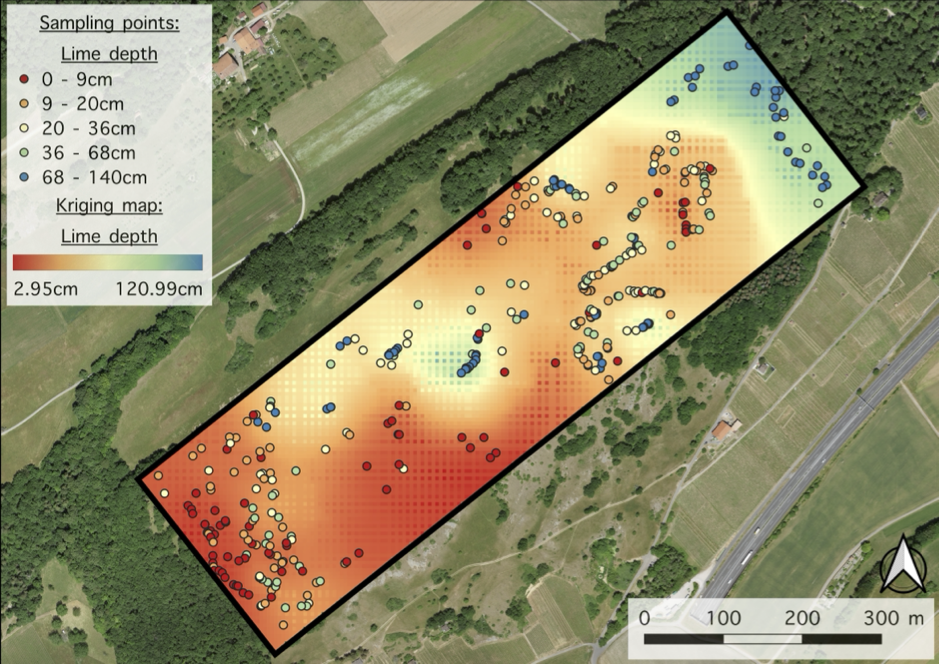
***

***Supplementary Figure 10: Interpolated topsoil pH (tph, 0-5 cm depth).*** *Sampling points are shown as circles on top of the interpolated map and colored according to field measurements. Background map: SWISSIMAGE 10 cm; ©swisstopo.*

*
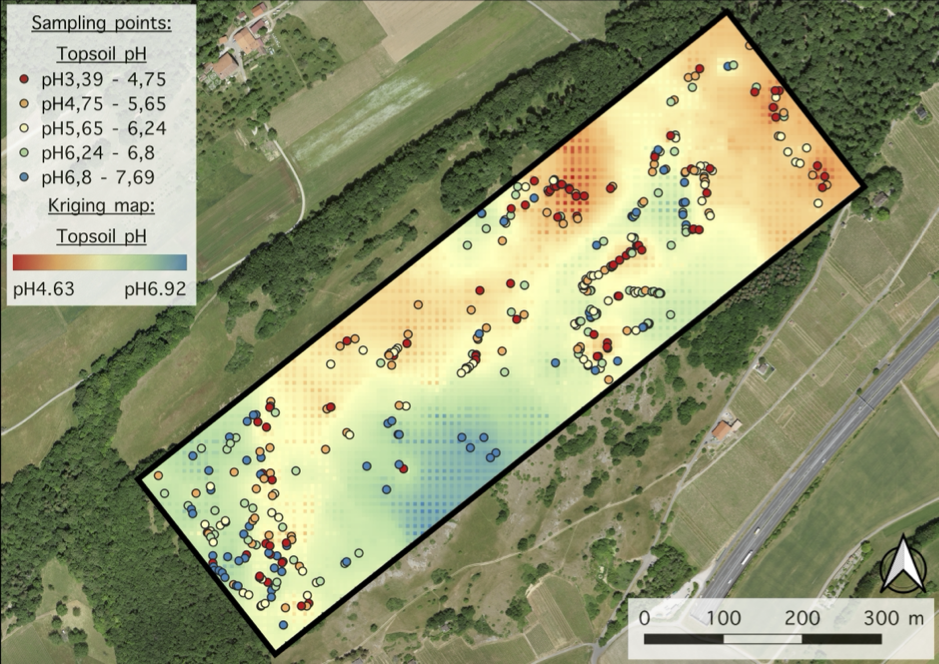
*

***Supplementary Figure 11: Interpolated deep soil pH (sph, 40-50 cm depth).***

*Sampling points are shown as circles on top of the interpolated map and colored according to field measurements. Background map: SWISSIMAGE 10 cm; ©swisstopo.*

***
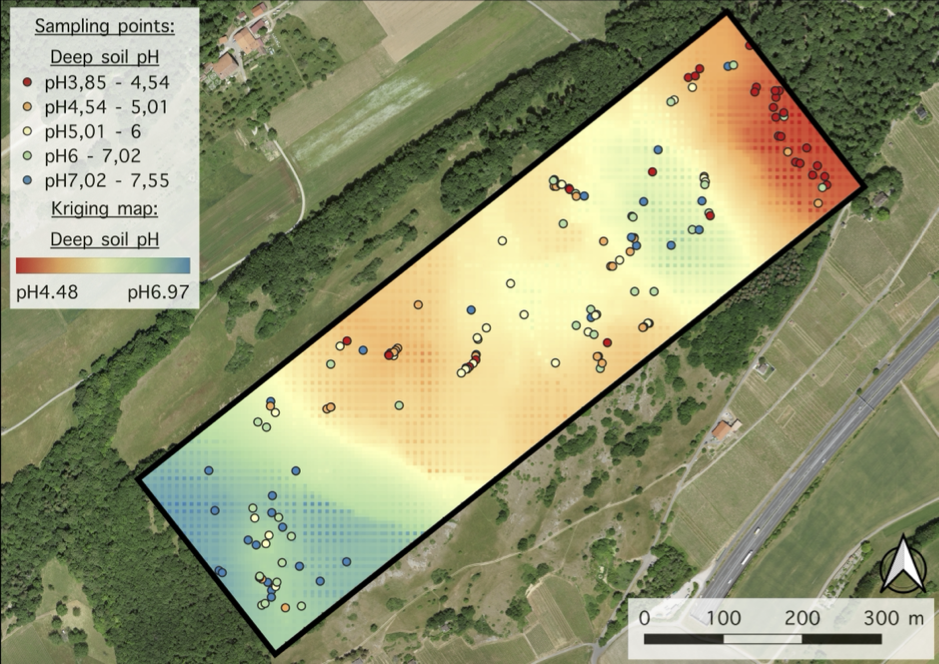
***

***Supplementary Figure 12: Results of the Bayesian Model Averaging.***

*Environmental variables (E-values) included in the top 100 models explaining the taxonomic proportion of Quercus pubescens for the full (top figure) and reduced (bottom figure) transect dataset. Explanatory variables are represented on the y-axis and cumulative model probabilities are represented on the x-axis. Models are ordered from left to right with decreasing posterior probabilities and variables are ordered from top to bottom with decreasing posterior model inclusion probabilities. The sign of the effect of each variable in a model is indicated by colour (purple: negative effect; yellow: positive effect). White colour indicates that the variable is not included in the model. Environmental variables that show a strong effect on the response variable (Q-values) are included in more models and in models with a higher posterior model probability. For details and abbreviations see Table 1 and Suppl. Table 2.*

******

***Supplementary Figure 13: Map indicating the spatial pattern of tree taxonomy and vertical curvature (vec).***

*The layer was processed using a Gaussian filtering with 6 m radius. Sampling points are colored according to the Quercus pubescens Q-values. Background map: SWISSIMAGE 10 cm; ©swisstopo.*

*
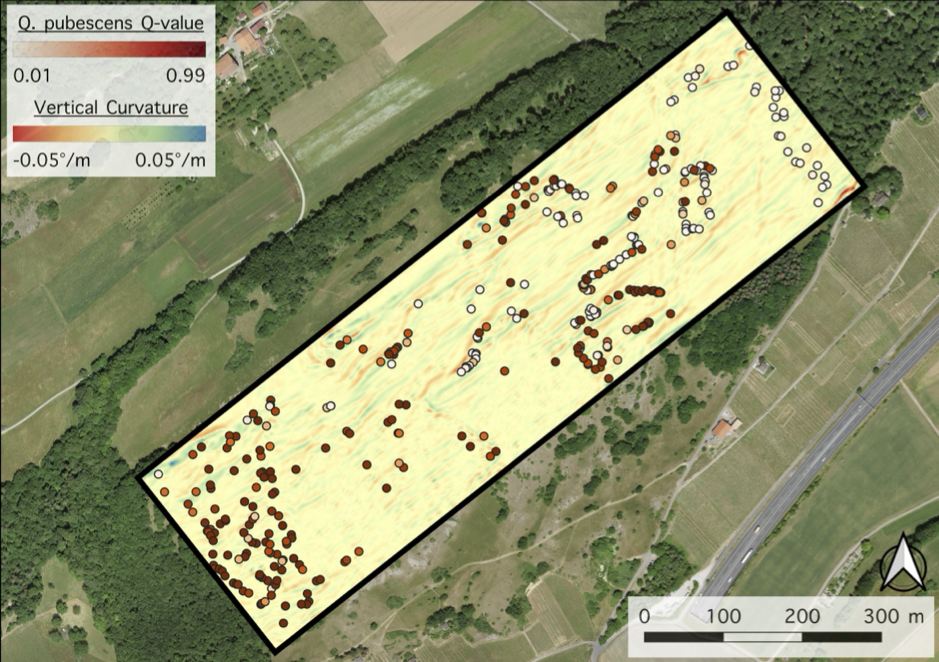
*

***Supplementary Figure 14: Map indicating spatial patterns of tree taxonomy and horizontal curvature (hoc).***

*The layer was processed using a Gaussian filtering with 6 m radius. Sampling points are colored according to the Quercus pubescens Q-values. Background map: SWISSIMAGE 10 cm; ©swisstopo.*

***
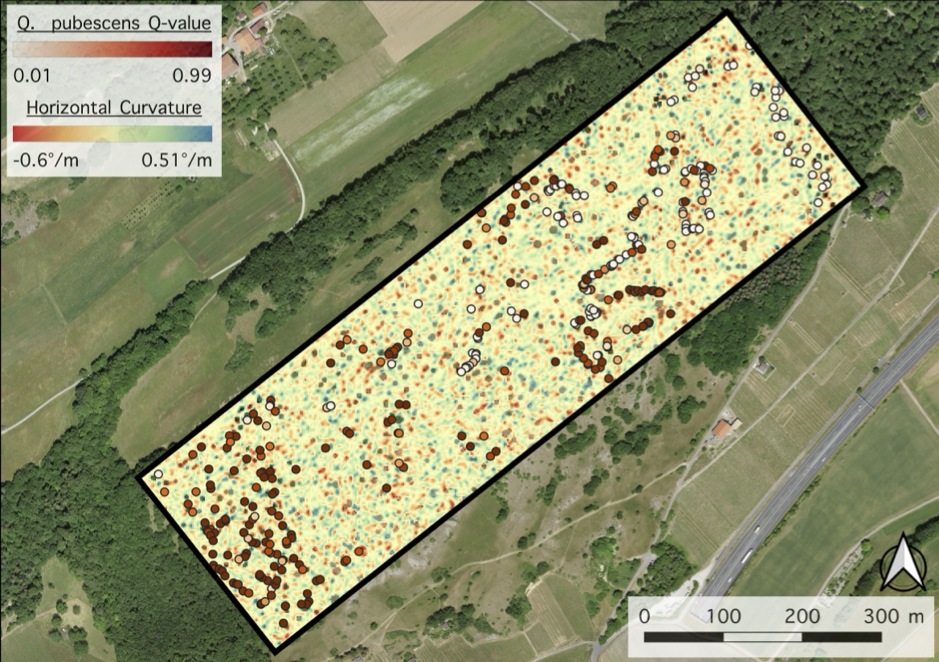
***

***Supplementary Figure 15: Map indicating spatial patterns of tree taxonomy and downslope distance gradient (ddg).****The layer was processed using a Gaussian filtering with 6 m radius. Sampling points are colored according to the Quercus pubescens Q-values. Background map: SWISSIMAGE 10 cm; ©swisstopo.*

***
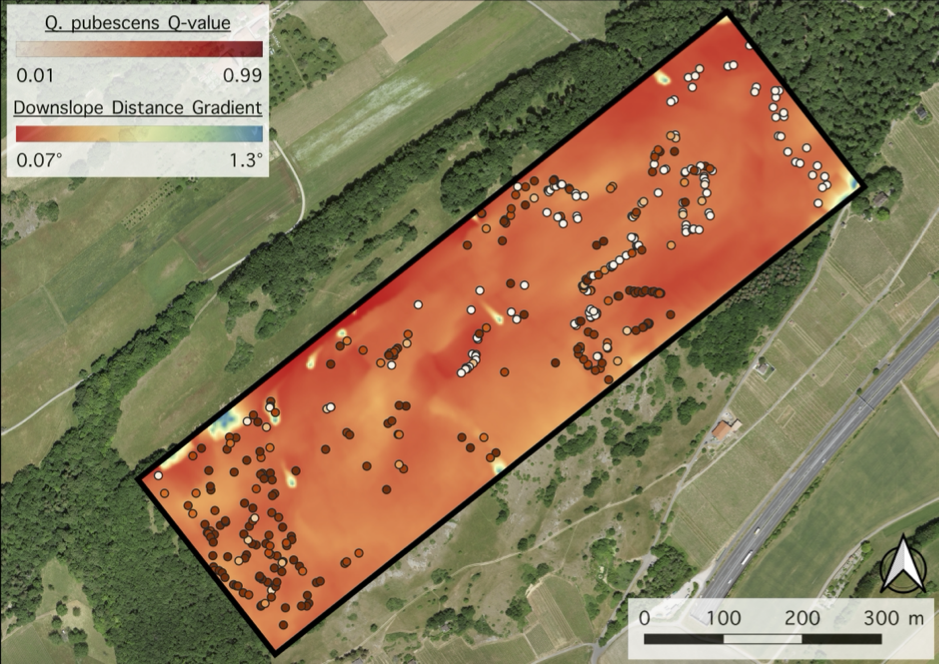
***

***Supplementary Figure 16: Map indicating spatial patterns of tree taxonomy and topographic positioning index (tpi).****The layer was processed using a Gaussian filtering with 6 m radius. Sampling points are colored according to the Quercus pubescens Q-values. Background map: SWISSIMAGE 10 cm; ©swisstopo.*

*
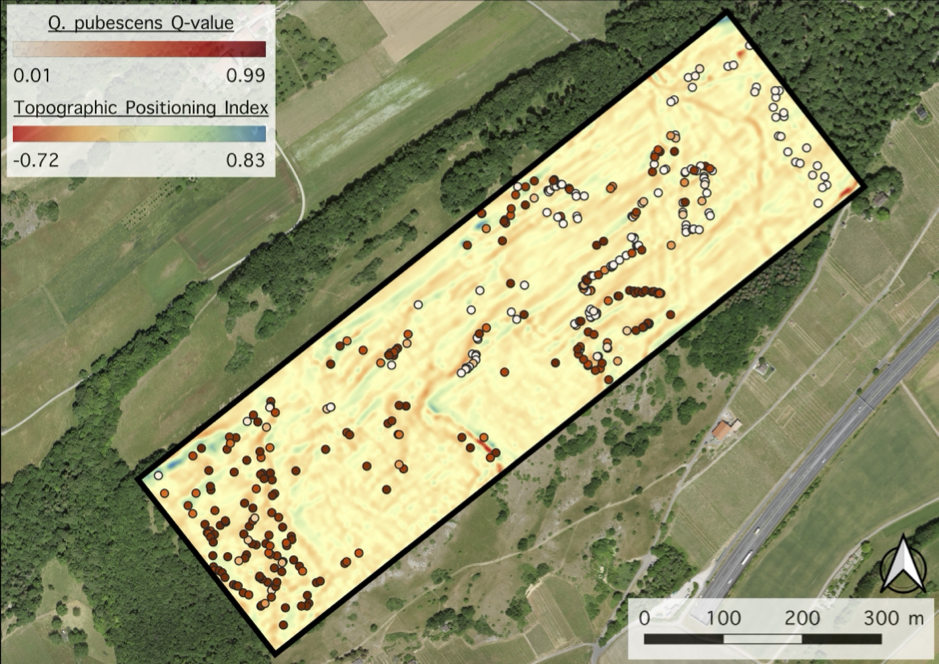
*

***Supplementary Figure 17: Map indicating spatial patterns of tree taxonomy and morphometric protection index (mpi).*** *The layer was processed using a Gaussian filtering with 6 m radius. Sampling points are colored according to the Quercus pubescens Q-values. Background map: SWISSIMAGE 10 cm; ©swisstopo.*

***
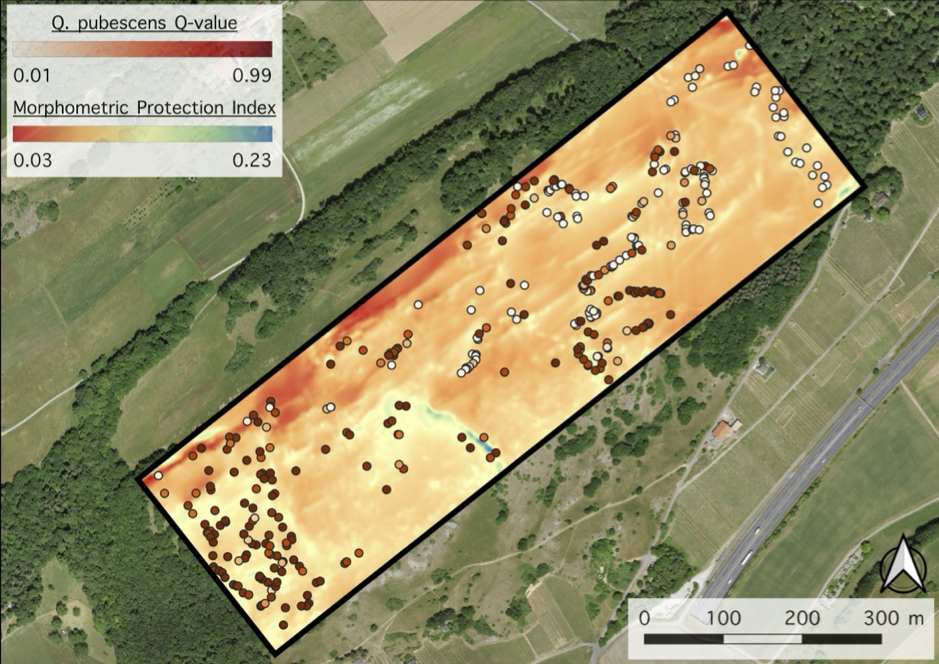
***

***Supplementary Figure 18: Map indicating spatial patterns of tree taxonomy and topographic wetness index (twi).***  *The layer was processed using a Gaussian filtering with 6 m radius. Sampling points are colored according to the Quercus pubescens Q-values. Background map: SWISSIMAGE 10 cm; ©swisstopo.*

***
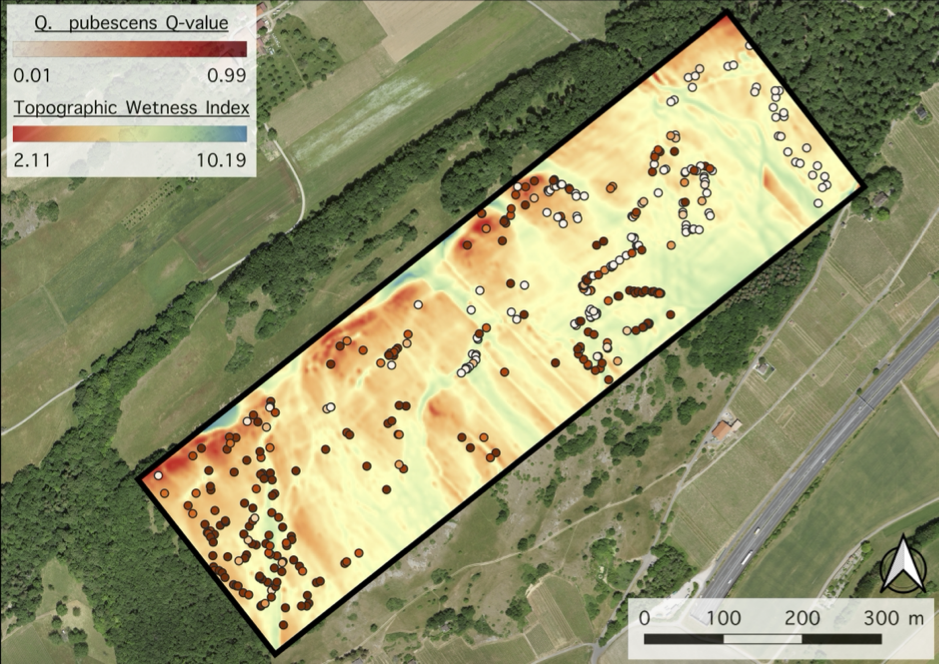
***

***Supplementary Figure 19: Map indicating spatial patterns of tree taxonomy and potential total insolation (pti).****The layer was processed using a Gaussian filtering with 6 m radius. Sampling points are colored according to the Quercus pubescens Q*-values. Background map: SWISSIMAGE 10 cm; ©swisstopo.

*
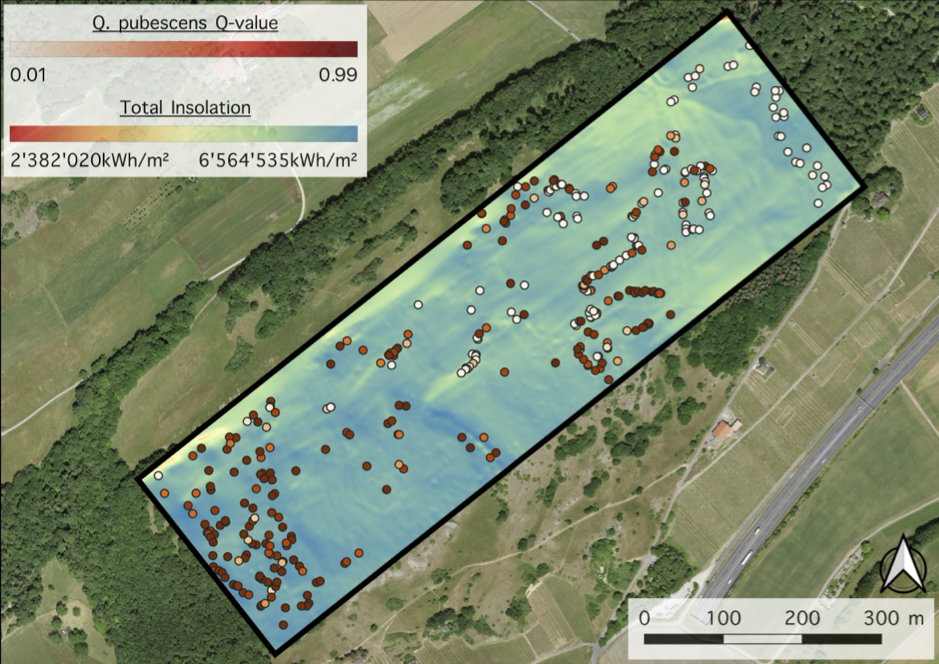
*

***Supplementary Figure 20: Mantel correlograms showing spatial autocorrelation of the taxonomic distances of trees, as well as euclidean distances of potential rooting depth (prd) and topsoil pH (tph).***

*Filled points indicate significant spatial autocorrelation of the three variables at the different distance classes.*

***Supplementary Table 1****:* ***SNP-marker set used for KASP genotyping.****The 50 best species-discriminating SNPs from Reutimann et al. (2020) based on a 5% threshold of allele frequency difference between the reference trees of any of the species pairs (indicated in the columns). Markers are ranked by frequency differences (in decreasing order) between the two study species Q. petraea und Q. pubescens.*

| **SNP marker** | ***Q. petraea* vs.  *Q. pubescens*** | ***Q. robur* vs.  *Q. petraea*** | ***Q. robur* vs.  *Q. pubescens*** |
| --- | --- | --- | --- |
| QuercusTarget094_409 | 0.626 | 0.840 | 0.213 |
| QuercusTarget123_78 | 0.521 | 0.022 | 0.543 |
| QuercusTarget043_370_v2 | 0.466 | 0.332 | 0.134 |
| QuercusTarget139_226 | 0.451 | 0.558 | 0.107 |
| QuercusTarget109_70 | 0.343 | 0.016 | 0.359 |
| QuercusTarget032_121 | 0.329 | 0.630 | 0.301 |
| QuercusTarget152_104 | 0.323 | 0.039 | 0.361 |
| QuercusTarget060_270 | 0.306 | 0.016 | 0.323 |
| QuercusTarget031_72 | 0.271 | 0.362 | 0.091 |
| QuercusTarget071_292 | 0.262 | 0.132 | 0.394 |
| QuercusTarget121_188 | 0.229 | 0.026 | 0.203 |
| QuercusTarget110_266 | 0.206 | 0.539 | 0.745 |
| QuercusTarget111_228 | 0.204 | 0.379 | 0.175 |
| QuercusTarget098_277 | 0.202 | 0.763 | 0.562 |
| QuercusTarget104_192 | 0.197 | 0.198 | 0.395 |
| QuercusTarget081_239 | 0.190 | 0.021 | 0.169 |
| QuercusTarget134_381 | 0.165 | 0.642 | 0.807 |
| QuercusTarget120_231 | 0.153 | 0.270 | 0.117 |
| QuercusTarget129_209 | 0.150 | 0.012 | 0.139 |
| QuercusTarget112_73 | 0.148 | 0.066 | 0.214 |
| QuercusTarget105_294 | 0.146 | 0.232 | 0.086 |
| QuercusTarget144_55 | 0.131 | 0.183 | 0.052 |
| QuercusTarget023_178 | 0.099 | 0.680 | 0.779 |
| QuercusTarget097_300 | 0.092 | 0.366 | 0.458 |
| QuercusTarget033_159 | 0.088 | 0.337 | 0.248 |
| QuercusTarget133_60 | 0.082 | 0.524 | 0.606 |
| QuercusTarget132_421 | 0.077 | 0.454 | 0.531 |
| QuercusTarget085_161 | 0.075 | 0.299 | 0.224 |
| QuercusTarget091_76 | 0.075 | 0.231 | 0.156 |
| QuercusTarget067_251 | 0.075 | 0.132 | 0.057 |
| QuercusTarget012_123 | 0.074 | 0.012 | 0.085 |
| QuercusTarget093_105 | 0.071 | 0.201 | 0.271 |
| QuercusTarget137_338 | 0.061 | 0.061 | 0.122 |
| QuercusTarget037_181 | 0.055 | 0.011 | 0.066 |
| QuercusTarget108_368 | 0.052 | 0.731 | 0.783 |
| QuercusTarget095_168 | 0.049 | 0.287 | 0.238 |
| QuercusTarget089_68 | 0.045 | 0.212 | 0.256 |
| QuercusTarget122_171_v2 | 0.042 | 0.686 | 0.728 |
| QuercusTarget107_347 | 0.025 | 0.125 | 0.101 |
| QuercusTarget106_89 | 0.022 | 0.178 | 0.200 |
| QuercusTarget151_295_v2 | 0.020 | 0.369 | 0.350 |
| QuercusTarget074_158 | 0.020 | 0.090 | 0.070 |
| QuercusTarget106_260 | 0.018 | 0.041 | 0.059 |
| QuercusTarget138_52 | 0.018 | 0.218 | 0.201 |
| QuercusTarget068_71 | 0.017 | 0.073 | 0.090 |
| QuercusTarget013_52 | 0.009 | 0.153 | 0.144 |
| QuercusTarget125_264 | 0.008 | 0.114 | 0.122 |
| QuercusTarget125_121 | 0.004 | 0.087 | 0.083 |
| QuercusTarget135_402 | 0.003 | 0.058 | 0.061 |
| QuercusTarget145_58 | 0.003 | 0.324 | 0.326 |

***Supplementary Table 2: Initial set of 17 environmental variables used in this study.***

| **Category** | **Abbreviation** | **Description** | **Unit** | **Range** | **Reference** |
| --- | --- | --- | --- | --- | --- |
| **Geographic**  **variables** | *lon* | Longitude (LV95) | m | 2,541,857.23 – 2,542,703.29 | - |
|  | *lat* | Latitude (LV95) | m | 1,188,288.98 – 1,189,020.76 | - |
| **Topographic variables** | *alt* | Altitude | m | 526.76 – 609.22 | - |
|  | *slp* | Slope | degrees | 1.53 – 25.53 | - |
|  | *nth* | Northness | - | -1.00 – -0.19 | Guisan et al. (1999) |
|  | *vec* | Vertical curvature | degrees m^-1^ | -0.03 – 0.03 | - |
|  | *hoc* | Horizontal curvature | degrees m^-1^ | -1.51 – 2.60 | - |
|  | *ddg* | Downslope distance gradient | degrees | 0.13 – 0.40 | Hjerdt et al. (2004) |
|  | *tri* | Terrain ruggedness index | - | 0.32 – 2.30 | Riley et al. (1999) |
|  | *tpi* | Topographic positioning index | - | -0.38 – 0.56 | Guisan et al. (1999) |
|  | *mpi* | Morphometric protection index | - | 0.04 – 0.15 | Yokoyama et al. (2002) |
|  | *twi* | Topographic wetness index | - | 2.48 – 8.49 | Böhner et al. (2002) |
|  | *pti* | Potential total insolation | kWh m^-2^ | 542,532,6.00 – 645,381,9.50 | Böhner & Antonić (2009) |
| **Soil**  **variables** | *prd* | Potential rooting depth | cm | 4.00 – 140.00 | - |
|  | *lid* | Lime depth | cm | 0.00 – 140.00 | - |
|  | *tph* | Top soil pH (0-5 cm) | - | 3.39 – 7.69 | - |
|  | *sph* | Deep soil pH (40-50 cm) | - | 3.85 – 7.55 | - |

**Appendix 1: Validation of taxonomic assignment**

To validate the Structure USEPOPINFO results, we analyzed the genetic data of the test and reference individuals with two additional approaches, a "basic" (naïve) Structure analysis and a support vector machine learning algorithm (SVM). The basic Structure algorithm was run with *K*=1-5 clusters using 10 iterations and 1,000,000 repetitions after a burn-in period of 100,000 runs. Results were summarized with Structure Harvester (Earl & vonHoldt, 2012) and re-ordered and averaged with Clumpak (Kopelman et al., 2015). The support vector machine (SVM) algorithm was run using the R-package e1071 (Meyer et al., 2023) in R 4.2.2 (R Development Core Team, 2022) and parameters from Reutimann et al. (2020). Support vector machine is an assumption-free model that assigns test individuals based on support vectors after being trained with a training set of reference individuals. The results (*Q*-values) of all three taxonomic assignment methods (Structure with USEPOPINFO, basic Structure, SVM) were compared using linear regressions.

*Q*-values of the Structure analysis with *K* = 3 using the USEPOPINFO parameter were confirmed by the two alternative taxonomic assignment methods, basic Structure (linear regression of *Q. pubescens* *Q*-values, R^2^ = 1, *p* < 0.001) and SVM (R^2^ = 0.91, *p* < 0.001).

***
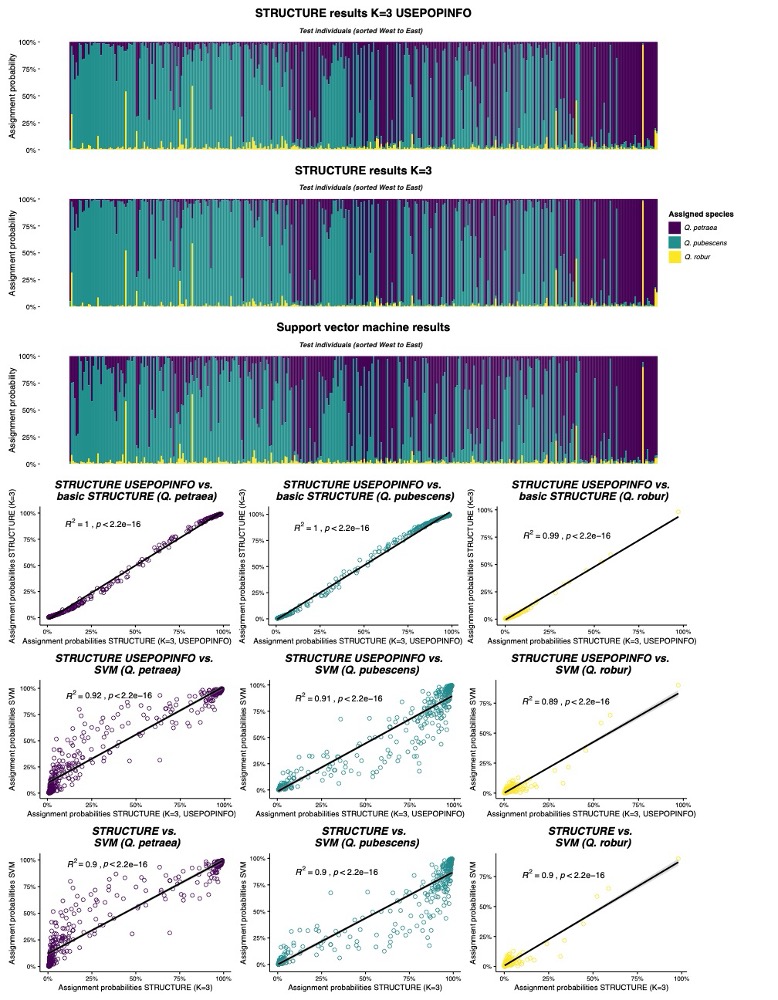
***

***Figure Appendix 1: Comparison of different assignment approaches.***

*Assignment probabilities from the different assignment approaches are shown as barplots (top). Each bar represents a single tree. The colors represent assignment probabilities to respective clusters (i.e. species). Reference individuals are excluded from the figure and the regression analyses. Test individuals (N = 380) are sorted from west to east. Scatter plots (bottom) show linear regressions of Q-values resulting from the different taxonomic assignment methods (basic STRUCTURE (K = 3), STRUCTURE with USEPOPINFO (K = 3), and Support Vector Machine (SVM, 3 groups), respectively.*

**Appendix 2: *Q*-value prediction**

The R-package GAMLSS (Stasinopoulos et al., 2023) was used to predict *Q-*values of *Q. pubescens* with a spline model based on the interaction of the two most important soil variables (*prd* and *tph*) resulting from the GLM and BMA analyses. Spline functions can describe complex relationships between variables by using piecewise-defined polynomial functions. Here, we specifically used a *lo* spline, which allows to create a loess fit surface based on one or more explanatory variables (Cleveland et al., 1992). In this case, additive loess fit models were created with three nodes using a LOGITNO family to account for proportional response variable data (Douma & Weedon, 2019): *Q*-value ~ potential rooting depth (degree 1) * topsoil pH (degree 1). A relatively low number of nodes (3) was chosen to predict a general trend in the response variable without implying a false accuracy and to avoid overfitting. Predicted *Q*-values results were visualized in a 3D plot using the *wireframe* function of the GAMLSS R-package.

***Figure Appendix 2: Wireframe plot of predicted Quercus pubescens Q-values.***

*Q-values (z-axis) were predicted based on the interaction of the two environmental variables topsoil pH (y-axis) and potential rooting depth (x-axis). Along the x-axis the wireframe surface is divided into 5 cm intervals, and, along the y-axis, the wireframe surface is divided into 0.1 pH intervals.*

The *Q*-value prediction using the two most important *in-situ* measured soil variables *tph* and *prd* summarizes and visualizes the previous analyses. The wireframe surface indicates that the *Q*-values of *Q. pubescens* increases with lower *prd* and higher *tph*. At the lower end of the pH-range, the *Q*-values did not exceed 0.4, independent of the soil depth. At the higher end of the soil depth range, the *Q*-values did not exceed 0.5 independent of the topsoil pH. These extreme combinations were present, but rather rare in the dataset.

**Supplementary literature cited**

Böhner, J., & Antonić, O. (2009). Land-surface parameters specific to topo-climatology. In T. Hengl & H. I. Reuter (Eds.), *Developments in soil science* (Vol. 33, pp. 195-226). Amsterdam, Netherlands: Elsevier Science.

Böhner, J., Koethe, R., Conrad, O., Gross, J., Ringeler, A., & Selige, T. (2002). *Soil regionalisation by means of terrain analysis and process parameterisation* (European Soil Bureau, Research Report 7).

Cleveland, W. S., Grosse, E., & Shyu, W. M. (1992). Local regression models. In *Statistical Models in S* (pp. 309-376). New York, USA: Routledge.

Douma, J. C., & Weedon, J. T. (2019). Analysing continuous proportions in ecology and evolution: A practical introduction to beta and Dirichlet regression. *Methods in Ecology and Evolution,* 10(9), 1412-1430.

Earl, D. A., & vonHoldt, B. M. (2012). STRUCTURE HARVESTER: a website and program for visualizing STRUCTURE output and implementing the Evanno method. *Conservation Genetics Resources,* 4(2), 359-361.

Guisan, A., Weiss, S. B., & Weiss, A. D. (1999). GLM versus CCA spatial modeling of plant species distribution. *Plant Ecology,* 143(1), 107-122.

Hjerdt, K. N., McDonnell, J. J., Seibert, J., & Rodhe, A. (2004). A new topographic index to quantify downslope controls on local drainage. *Water Resources Research,* 40(5), W05602.

Kopelman, N. M., Mayzel, J., Jakobsson, M., Rosenberg, N. A., & Mayrose, I. (2015). CLUMPAK: a program for identifying clustering modes and packaging population structure inferences across K. *Molecular Ecology Resources,* 15(5), 1179-1191.

Meyer, D., Dimitriadou, E., Hornik, K., Weingessel, A., Leisch, F., & Lin, C.-C. (2023). e1071: Misc functions of the department of statistics: TU Wien. Retrieved from <https://cran.r-project.org/web/packages/e1071/index.html>

R Development Core Team. (2022). *R: a language and environment for statistical computing*. Retrieved from <http://www.R-project.org>

Reutimann, O., Gugerli, F., & Rellstab, C. (2020). A species-discriminatory single-nucleotide polymorphism set reveals maintenance of species integrity in hybridizing European white oaks (*Quercus* spp.) despite high levels of admixture. *Annals of Botany,* 125(4), 663-676.

Riley, S. J., DeGloria, S. D., & Elliot, R. (1999). A terrain ruggedness index that quantifies topographic heterogeneity. *Intermountain Journal of Sciences,* 5(1-4), 23-27.

Stasinopoulos, M., Rigby, B., Voudouris, V., Akantziliotou, C., Enea, M., & Kiose, D. (2023). gamlss: Generalised additive models for location scale and shape. Retrieved from <https://cran.r-project.org/web/packages/gamlss/index.html>

Yokoyama, R., Shirasawa, M., & Pike, R. J. (2002). Visualizing topography by openness: A new application of image processing to digital elevation models. *Photogrammetric Engineering and Remote Sensing,* 68(3), 257-265.
